# Metagenomic sequencing reveals viral diversity of mosquitoes from Egypt: co-circulation of multiple insect-specific viruses

**DOI:** 10.1128/spectrum.02135-25

**Published:** 2026-03-06

**Authors:** Shenglin Chen, Yuanyuan Li, Jinbo Xue, Yuwan Hao, Mona G. A. Shaalan, Enas H. S. Ghallab, Zhaoyu Guo, Shuqing Jin, Yuan Fang, Emad I. M. Khater, Shizhu Li

**Affiliations:** 1 National Institute of Parasitic Diseases, Chinese Center for Disease Control and Prevention; Chinese Center for Tropical Diseases Research; National Key Laboratory of Intelligent Tracking and Forecasting for Infectious Diseases; NHC Key Laboratory of Parasite and Vector Biology; WHO Collaborating Centre for Tropical Diseases; National Center for International Research on Tropical Diseases, Ministry of Science and Technology 308176, Shanghai, China; 2 Deptartment of Entomology, Faculty of Science, Ain Shams University, Cairo, Egypt; 3 Huangpu District Center for Disease Prevention and Control (Huangpu District Health Supervision Institute), Shanghai, China; 4 School of Global Health, Chinese Center for Tropical Diseases Research-Shanghai Jiao Tong University School of Medicine 674520, Shanghai, China; Chinese Academy of Sciences Wuhan Institute of Virology, Wuhan, China

**Keywords:** Metagenomic analysis, mosquito viromes, *Culex flavivirus*, insect specific flavivirus, putative novel virus

## Abstract

Mosquito-borne virus surveillance is pivotal for investigating mosquito viromes, facilitating understanding of viral evolutionary histories and genetic diversity. Natural viral communities in mosquitoes include not only insect-specific viruses (ISVs) but also viruses infecting symbiotic microorganisms. In this study, a total of 654 mosquito samples—encompassing species from the *Aedes* and *Culex* genera—were collected from Egypt and subjected to metagenomic sequencing analysis. Over 130 virus species were identified, grouped into 35 families or equivalent taxonomic ranks. Detected ISVs included Culex flavivirus (CxFV), Kustavi Toti-like virus, Hanko Toti virus 5, Culex phasma-like virus (CPLV), Culex Iflavi-like virus 1, Culex Iflavi-like virus 4, Guadeloupe Culex rhabdovirus (GCRV), and Sarawak virus, confirming concurrent ISV circulation in Egyptian mosquitoes. Phylogenetic analyses of these ISVs revealed their closest evolutionary affinities to viral genome sequences originating from the Middle East, Europe, Oceania, and Asia. Specifically, Egyptian CxFV strains exhibited a closer genetic relationship with the tropical lineage within the Latin American/Caribbean/Africa genotype. Furthermore, our study uncovered 10 putative novel viruses, which are distributed across seven viral families: Amagaviridae, Chrysoviridae, Mitoviridae, Totiviridae, Virgaviridae, Narnaviridae, and Orthomyxoviridae. Collectively, our findings emphasize the necessity for more in-depth investigations into arthropod viromes—encompassing both mosquitoes and ticks—in Egypt, as well as in neighboring African and Middle Eastern countries. Such research is critical for enhancing our understanding of viral diversity and evolutionary biology, elucidating their roles in mosquito–pathogen–host interactions, and exploring their potential as biocontrol agents against vector-borne diseases of public health importance.

**IMPORTANCE:**

Mosquito-borne viruses are estimated to cause over 100 million human infections annually, making surveillance of these pathogens increasingly crucial amid growing international travel and trade. Egypt, situated in northeastern Africa, serves as a geopolitical and geographical hub connecting Asia, Europe, and Africa—a unique location that complicates the surveillance of mosquito-borne viruses. Arboviruses persist in nature through cyclical transmission between arthropod vectors (e.g., mosquitoes, ticks, and midges) and susceptible vertebrate hosts. Despite this, systematic investigations into mosquito viromes remain relatively scarce in Egypt. The present study aimed to explore the genetic diversity and evolutionary relationships of mosquito-associated viruses in Egypt using metaviromic sequencing. Our findings significantly expand the current knowledge of both known and previously uncharacterized mosquito-associated viruses in the region, while also providing complete genome sequences of several viruses that may infect arthropods or vertebrates, and potentially interfere with the replication of pathogenic arboviruses.

## INTRODUCTION

Mosquitoes transmit a diverse array of arboviral diseases, posing substantial threats to global public health. It is estimated that mosquito-borne viruses cause over 100 million human infections annually worldwide, with the tropical regions bearing a disproportionate burden (1). As primary vectors of arboviruses, mosquitoes are responsible for transmitting several high-impact pathogens, including those causing dengue, Zika, West Nile, and Rift Valley fevers (2). Owing to their pivotal roles in viral ecology and the transmission of mosquito-borne diseases (MBDs), mosquitoes have emerged as key surveillance targets for infectious disease prevention and control. Arboviruses persist in nature through cyclical transmission between arthropod vectors—such as mosquitoes, ticks, and midges—and susceptible vertebrate hosts (3). To date, more than 130 arboviruses have been recognized as pathogenic to humans and/or other vertebrates (4, 5). The rapid advancement of high-throughput sequencing technology has facilitated the identification of a vast number of arthropod-borne viruses (ABVs), including a multitude of insect-specific viruses (ISVs) that exhibit no known vertebrate tropism (6).

Egypt, located in northeastern Africa, serves as a critical geographic crossroads connecting Asia, Europe, and Africa. This unique positional advantage, however, complicates the surveillance of mosquito-borne viruses (MBVs) within its borders (7). At least five clinically significant MBVs have been documented in Egypt: Dengue Fever virus (DENV), Sindbis virus (SINV), West Nile virus (WNV), Rift Valley Fever virus (RVFV), and Chikungunya virus (CHIKV) (8). Climatologically, Egypt is characterized by a tropical Mediterranean climate along its northern coast and a tropical desert climate across the remainder of the country. Several factors converge to hinder effective control of DENV, RVFV, and WNV in the region: the presence of competent *Culex* vector species, the recent establishment of invasive *Aedes aegypti* populations, increased maritime trade and human mobility through Egyptian territories, and ongoing political instability in neighboring regions (8). Despite these pressing concerns, to the best of our knowledge, no comprehensive studies have systematically characterized the diversity of arboviruses carried by field mosquito populations in Egypt. Accordingly, the present study aimed to characterize the RNA viromes associated with medically important mosquito species collected from three key regions of Egypt—the Greater Cairo and Nile Valley-Delta, the Nile Valley-Upper Egypt, and the Red Sea—and to elucidate the molecular and phylogenetic relationships of the identified viruses.

With the continuous advancement of high-throughput sequencing technology and declining sequencing costs, metagenomic sequencing has emerged as a powerful non-targeted tool ideally suited for high-throughput viral discovery. It has become indispensable for delineating the viral spectra carried by mosquito vectors, identifying previously unrecognized viruses, and quantifying the diversity and abundance of mosquito-borne pathogens (9–11). Consequently, MBV surveillance leveraging meta-viromic sequencing to probe mosquito viromes is crucial for understanding viral evolutionary histories and diversity—particularly for the discovery of novel arboviruses.

Building on these objectives, this study employed meta-viromic sequencing to investigate the diversity, abundance, and regional variations of RNA viromes in mosquitoes across the three aforementioned Egyptian regions. Additionally, through comprehensive detection, annotation, and phylogenetic analysis, we sought to identify putative novel arboviruses in field mosquito populations. The findings of this study provide valuable insights to inform surveillance efforts for mosquito-associated viruses in Egypt, with implications for biodiversity conservation, public health, and veterinary medicine.

## MATERIALS AND METHODS

### Survey area and mosquito collection

Routine mosquito surveillance is ongoing in strategically important areas of Egypt, with the primary objective of collecting *Culicidae* mosquitoes for viral characterization and detection. Special attention is focused on arboviruses such as DENV, RVFV, and SINV—pathogens that can trigger severe disease outbreaks and impose substantial public health burdens if not identified during the early stages of mosquito infection. Adult mosquitoes were collected using three methods: CDC light traps, manual aspiration from human landing catches, and the spray-sheet technique. Mosquito sample collection, transportation, and subsequent taxonomic and molecular identification were conducted following previously established protocols (12).

For this preliminary investigation of mosquito-associated viruses, a total of 654 mosquitoes were collected from governorates spanning three key regions: the Greater Cairo and Nile Valley-Delta (Cairo, Giza, Qalyubia, and Faiyum), the Nile Valley-Upper Egypt (Aswan), and the Red Sea (Red Sea Governorate, Hurghada city). Detailed survey sites are illustrated in Supplementary Map (Fig. S1) and summarized in Table S1. Considering factors such as mosquito species and sampling location, 654 mosquitoes were divided into 14 mosquito pools, which, in turn, were used for the meta-viromic sequencing study.

### RNA extraction

Mosquito tissue homogenization was performed at 4°C using a Jingxin Mixer Mill (Jingxin, Shanghai, China)—a fully automated high-throughput sample grinder—operated at 25 Hz for 3 min. Following homogenization, TRIzol reagent (Invitrogen, Carlsbad, CA, USA) was added to each sample pool, and the mixture was centrifuged at maximum speed at 4°C to separate phases. The supernatant from each processed sample was transferred to a dedicated processing cartridge and loaded onto the MagNA Pure 96 system (Roche, Basel, Switzerland) for automated RNA extraction. RNA extraction was specifically carried out using the MagNA Pure 96 Cellular RNA High Volume Kit (Roche), which includes a DNase treatment step to ensure the recovery of DNA-free RNA, strictly following the manufacturer’s standard operating procedures.

### Target gene sequencing

First-strand cDNA synthesis was performed via reverse transcription-polymerase chain reaction (RT-PCR) using the Takara PrimeScript RT Reagent Kit with gDNA Eraser (TAKARA BIO, Shiga, Japan), a reagent system designed to eliminate genomic DNA contamination prior to reverse transcription. To verify RNA integrity in each mosquito pool, PCR amplification targeting the mosquito 18S rRNA gene was conducted using the primer pair 18S417 and 18S920c (13). Additionally, a panel of PCR assays was employed to detect specific viral genes, as previously reported: the partial NS5 gene of flaviviruses (14), the NSP1 gene of alphaviruses (15), and the S segment of bunyaviruses (16).

### Library construction and meta-viromic sequencing

Ribosomal RNA (rRNA) depletion was first performed using the Ribo-off rRNA Depletion Kit (Vazyme, Cat. No. N406) to enrich viral RNA in the samples. The processed RNA was then fragmented, followed by reverse transcription to synthesize first-strand cDNA and subsequent synthesis of double-stranded cDNA. RNA libraries were constructed using the ALFA-SEQ DNA Library Prep Kit (Illumina-compatible), and high-throughput sequencing was performed on the Illumina NovaSeq platform with 150 bp paired-end (PE150) read generation.

### Bioinformatics analysis

Raw sequencing data underwent quality control (QC) using Trimmomatic software (17). Subsequently, sequences derived from ribosomal RNA, host genomes, and bacteria were filtered out using BBMap (https://sourceforge.net/projects/bbmap), yielding a high-quality set of clean reads. *De novo* assembly of these clean reads was performed using two robust assemblers—SPAdes (https://github.com/ablab/spades) and SOAPdenovo-Trans (https://github.com/aquaskyline/SOAPdenovo-Trans)—which efficiently reconstruct contiguous sequences (contigs) from fragmented data. To identify viral species and infer their evolutionary relationships, the assembled contigs were systematically aligned against the Virus-NT database using BLAST. Viral coverage of mosquito viromes at the pool level was calculated as previously described (18), defined as the percentage of viral reads relative to the total raw reads. Raw reads herein refer to the paired-end sequences generated post-library sequencing prior to bioinformatics processing.

Virus identification and taxonomic annotation were conducted using CheckV software (19), with viral species confirmation based on the annotation information of CheckV’s target reference sequences in the database. Further validation of viral contig classification was performed via BLASTx and BLASTn alignments against the non-redundant nucleotide (nt) and non-redundant protein (nr) databases, respectively (20). Novel virus species were delineated based on nucleotide (nt) and amino acid (aa) sequence identities, adhering to the species demarcation criteria established by the International Committee on Taxonomy of Viruses (ICTV; https://talk.ictvonline.org/, accessed March 2025). For genera lacking explicit ICTV criteria, we applied a threshold of <90% aa identity in the RNA-dependent RNA polymerase (RdRp) domain or <80% nt identity across the complete genome relative to known viruses.

Gene prediction for viral contigs was performed using Prokka (v1.13) (21), followed by filtering of contigs shorter than 500 bp. The number and length of predicted genes were quantified for meta-viromic analysis. Protein sequences of the predicted genes were aligned against viral sequences in the UniProtKB/Swiss-Prot database (ViralZone, reviewed proteins; https://viralzone.expasy.org/) using BLASTp (v2.9.0+) (22), with a threshold of e-value <1e-3 for significant hits. Viruses identified in this study that have not been isolated and cultured are designated as “putative novel viruses.” These viruses were named “Egypt mosquito virus” with appended family information and GenBank accession numbers.

### Statistical analysis

Viral abundance was quantified using the RPKM (reads per kilobase per million reads) metric (23). Mosquito pools were stratified into three groups based on sampling regions: the GC group (Greater Cairo and Nile Valley-Delta), encompassing pools from Cairo, Giza, Qalyubia, and Faiyum; the AS group (Nile Valley-Upper Egypt), consisting of pools from Aswan; and the RS group (Red Sea), including pools from the Red Sea Governorate (Hurghada). We analyzed the relative abundance percentage of RPKM values at both the viral family and species levels across the three groups. Differences in the Shannon diversity index among groups were tested using the Kruskal–Wallis test, followed by post-hoc multiple comparisons via the Nemenyi test with *P*-value correction. Principal Coordinate Analysis (PCoA) was performed to assess variations in viral composition among groups based on Bray–Curtis dissimilarities. Permutational Multivariate Analysis of Variance (PERMANOVA) was applied to beta diversity data to determine statistical differences, with PERMANOVA tests for distance matrices generated using the *adonis2* function in the R package Vegan (both overall and pairwise comparisons). Linear Discriminant Analysis Effect Size (LEfSe) was used to identify viral biomarkers, with analyses conducted via the online Galaxy platform (http://huttenhower.sph.harvard.edu/lefse/). A linear discriminant analysis (LDA) score threshold of 2.0 and *P* < 0.05 was considered indicative of significant biomarkers. All statistical tests were two-tailed, with *P* < 0.05 defined as statistically significant. All analyses were performed using R software (version 4.1.0; R Foundation for Statistical Computing, Vienna, Austria).

### Phylogenetic analysis

Multiple sequence alignments were constructed using ClustalW2 (24) with default parameters, incorporating viral genomes identified in this study and reference sequences retrieved from NCBI. Alignments were manually adjusted where necessary to optimize accuracy. Reference sequences were selected to represent diverse geographic origins (countries and provinces) and host species. Phylogenetic trees were constructed for the identified viruses using MEGA v11.0 software (25). Neighbor-joining trees were generated based on the p-distance method (26), with branch support evaluated via 1000 bootstrap replications.

## RESULTS

### Metagenomic analysis of mosquitoes in Egypt

A total of 654 mosquitoes were collected from 6 governorates in Egypt, representing three species: *Aedes caspius*, *Culex perexiguus*, and the *Cx. pipiens* complex (Table S1). These mosquitoes were grouped into 14 pools based on 3 criteria: sex, species, and sampling location. Metagenomic sequencing of the 14 pools generated 677,113,086 paired-end raw reads. After quality control (QC) filtering and removal of host-derived sequences, 61,938,054 high-quality clean reads were retained for downstream analyses. Ultimately, we identified 3,139 viral contigs, which were classified into 35 viral families or unclassified viral groups. The average viral coverage of mosquito viromes across mixed-genus pools was 9.15%, with individual coverage values for each of the 14 pools provided in Table S1.

**Fig 1:**
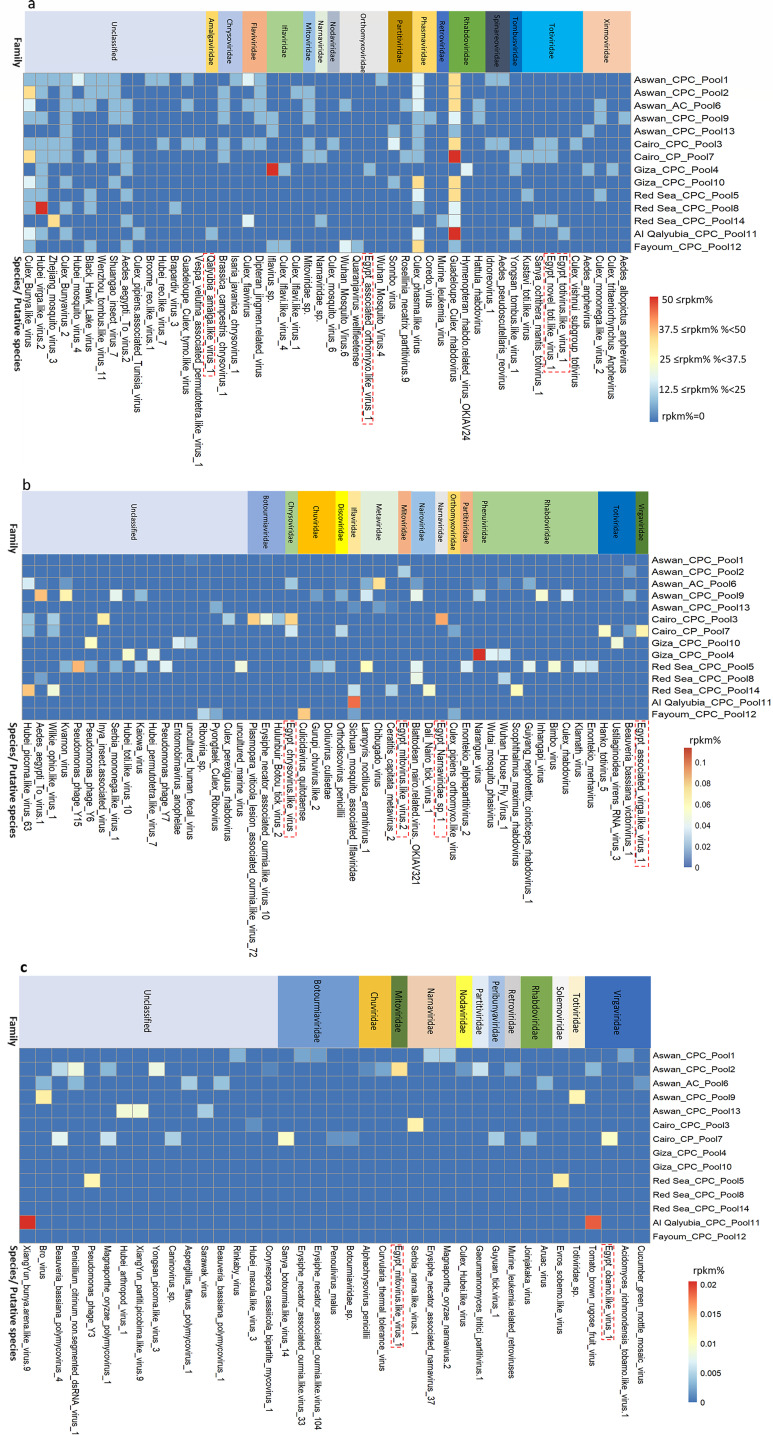
Heatmap depicting viral abundance at the species or equivalent taxonomic rank. (**a**) Top 50 viruses ranked by total relative abundance (RPKM percentage, summed across 14 mosquito pools). (**b**) Viruses ranked 51–100 by total relative abundance (RPKM percentage, summed across 14 mosquito pools). (**c**) Viruses ranked 101–139 by total relative abundance (RPKM percentage, summed across 14 mosquito pools). Heatmaps were generated based on RPKM-derived relative abundance percentages. Species enclosed in red boxes indicate putative novel viruses identified in this study. Abbreviations: RPKM = reads per kilobase of transcript per million mapped reads; CPC = *Culex pipiens* complex; AC = *Aedes caspius*; CP = *Cx* perexiguus.

This study uncovered more than 130 virus species, distributed across 35 taxonomic clusters (families or equivalent ranks) (Fig. 1). Viruses belonging to the families Rhabdoviridae, Phasmaviridae, Narnaviridae, Partitiviridae, Tombusviridae, and Totiviridae exhibited broad distribution across the Egyptian mosquito sample pools (Fig. S2). Notably, five viruses—Guadeloupe Culex rhabdovirus (GCRV), Culex phasma-like virus (CPLV), Culex Bunya-like virus, Culex Bunyavirus 2, and Hubei Virga-like virus—were detected in nearly all mosquito sample pools (Fig. 1). Viral contig composition varied among mosquito species-specific pools: *Culex* pools: A large proportion of viral contigs were affiliated with the families Rhabdoviridae, Flaviviridae, Iflaviridae, and Phasmaviridae (Fig. S2), species-level analysis revealed these contigs primarily corresponded to GCRV, Culex flavivirus (CxFV), *Iflavirus* sp., and CPLV (Fig. 1); *Aedes caspius* pool: Dominant viral contigs were derived from Rhabdoviridae, Flaviviridae, and Phasmaviridae (Fig. S2), with species-level assignments focusing on GCRV, CxFV, and CPLV (Fig. 1); *Cx. perexiguus* pool: high proportions of contigs belonged to Rhabdoviridae and Totiviridae, predominantly corresponding to GCRV and Kustavi Toti-like virus (Fig. S2). Figure S2 provides a comprehensive overview of all RNA viruses detected via meta-viromic sequencing, categorized at the family level. Further analysis of viral distribution across pools showed that 31.4% of viruses (at the family level or equivalent rank) were shared among the three mosquito species pools (Fig. S3a). In contrast, only 8.6% of viruses were common across the pools when analyzed at the species level (Fig. S3b).

### Phylogenetic analysis

PCR screening identified one strain of Culex flavivirus (CxFV) from a wild *Cx. pipiens* complex mosquito collected in the Red Sea region. Phylogenetic analysis of a partial NS5 gene fragment revealed that this Egyptian CxFV strain shared the closest evolutionary relationship with a CxFV isolate from *Cx. quinquefasciatus* in Jeddah, Saudi Arabia, with 98.31% nucleotide homology (Fig. S4). Subsequently, the nearly complete genome sequence of this Egyptian CxFV strain was acquired through meta-viromic sequencing (GenBank Accession No.: PV585599).

**Fig 2:**
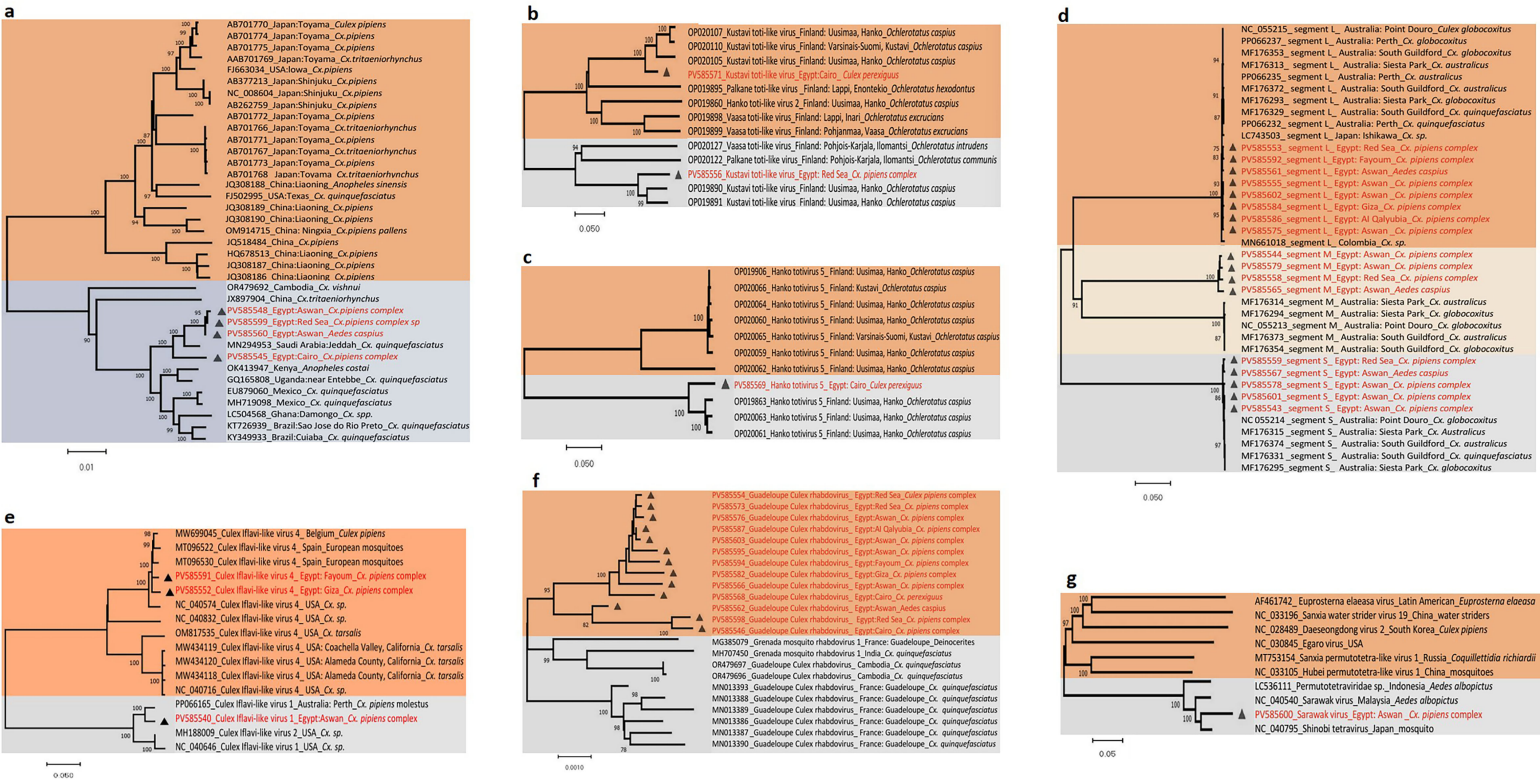
Phylogenetic analyses of viruses from six families identified by meta-viromic sequencing of mosquito samples in Egypt, including Culex flavivirus (CxFV, Flaviviridae), Kustavi toti-like virus (Totiviridae), Hanko totivirus 5 (Totiviridae), Culex phasma-like virus (CPLV, Phasmaviridae), Culex iflavi-like virus 1, Culex iflavi-like virus 4 (Iflaviridae), Guadeloupe Culex rhabdovirus (GCRV, Rhabdoviridae), and Sarawak virus (Alphatetraviridae). (**a**) Phylogenetic tree of CxFV genomes generated from sequences obtained in this study and reference sequences retrieved from the NCBI GenBank database. (**b**) Phylogenetic tree of Kustavi toti-like virus based on genomic sequences from this study and NCBI GenBank references. (**c**) Phylogenetic tree of Hanko totivirus 5 constructed using genomic sequences from this study and NCBI GenBank references. (**d**) Phylogenetic tree of CPLV genomic segments (L, M, and S) derived from sequences in this study and NCBI GenBank references. (**e**) Phylogenetic tree of Culex iflavi-like virus 1 and Culex iflavi-like virus 4 based on genomic sequences from this study and NCBI GenBank references. (**f**) Phylogenetic tree of GCRV genomes generated from sequences obtained in this study and NCBI GenBank references. (**g**) Phylogenetic tree of Sarawak virus genomic sequences derived from this study and NCBI GenBank references. Bootstrap values (1,000 replicates; not shown for values < 75%) from the neighbor-joining method are indicated above major lineages. Triangles denote sequences generated in this study.

CxFV sequences were further detected in four mosquito pools via meta-viromic sequencing, encompassing both *Cx. pipiens* complex and *Ae. caspius* specimens collected from Aswan, Cairo, and the Red Sea Governorate (RSG). These Egyptian CxFV sequences exhibited the highest sequence similarity to CxFV strains isolated from *Cx. quinquefasciatus* in Saudi Arabia (Fig. 2a). A phylogenetic tree constructed using the nearly complete genome sequences of 37 CxFV strains (Fig. 2a) resolved two well-supported monophyletic clades. Clade 1 included CxFV sequences from Asia (China and Japan) and the United States, while Clade 2 comprised CxFV strains from the Caribbean, Africa, and Latin America. Combined sequence analysis and phylogenetic characterization confirmed that all CxFV sequences identified in this study fell within Clade 2.

Kustavi Toti-like virus was detected in two pools containing *Cx. perexiguus* and *Cx. pipiens* complex mosquitoes, sampled from the RSG (Hurghada) and the Greater Cairo region. These Egyptian sequences were most closely related to a Kustavi Toti-like virus isolate from *Ochlerotatus caspius* in Finland (Fig. 2b). Additionally, Hanko Totivirus 5 was identified in a *Cx. perexiguus* pool from the Greater Cairo region, sharing 90.72% sequence identity with a Hanko Totivirus 5 strain from *Oc. caspius* in Finland (GenBank Accession No.: OP019863) (Fig. 2c). The genome segments (L, M, and S) of Culex phasma-like virus (CPLV) were detected in *Cx. pipiens* complex and *Ae. caspius* pools collected from Aswan, Giza, Cairo, Qalyubia, Faiyum, and the RSG. These Egyptian CPLV sequences showed the closest evolutionary affinity to CPLV strains from Australia (Fig. 2d). Two iflavirus species were identified in *Cx. pipiens* complex pools from Aswan (Fig. 2e): (i) Culex Iflavi-like virus 1, which shared 96.45% sequence identity with a Culex Iflavi-like virus 1 isolate from the *Cx. pipiens* complex in Australia (GenBank Accession No.: PP066165) and (ii) two genome sequences of Culex Iflavi-like virus 4, which were most closely related to a Culex Iflavi-like virus 4 isolate from mosquitoes in Spain. Notably, GCRV genome sequences were detected in nearly all tested mosquito pools. Notably, GCRV genome sequences were detected in nearly all tested mosquito pools. Phylogenetic analysis clustered all Egyptian GCRV sequences within a single major clade (Fig. 2f); although this clade was closely related to a GCRV strain from *Cx. quinquefasciatus* in France, it formed a distinct subclade. Furthermore, a genomic sequence of Sarawak virus was identified in the *Cx. pipiens* complex from Aswan, which showed the closest relationship to Sarawak virus from Malaysia (GenBank Accession No.: NC_040540, percentage identity: 90.66%) (Fig. 2g).

Furthermore, this study obtained 20 genomic sequences of ISVs that remain unclassified at the taxonomic level; these include 9 sequences of Culex Bunyavirus 2, 2 of Guadeloupe Culex tymo-like virus, 7 of Hubei virga-like virus 2, 1 of Iflavirus sp., and 1 of Culex pipiens-associated Tunisia virus.

### Annotation and phylogenetic analysis of putative novel viruses

**Fig 3:**
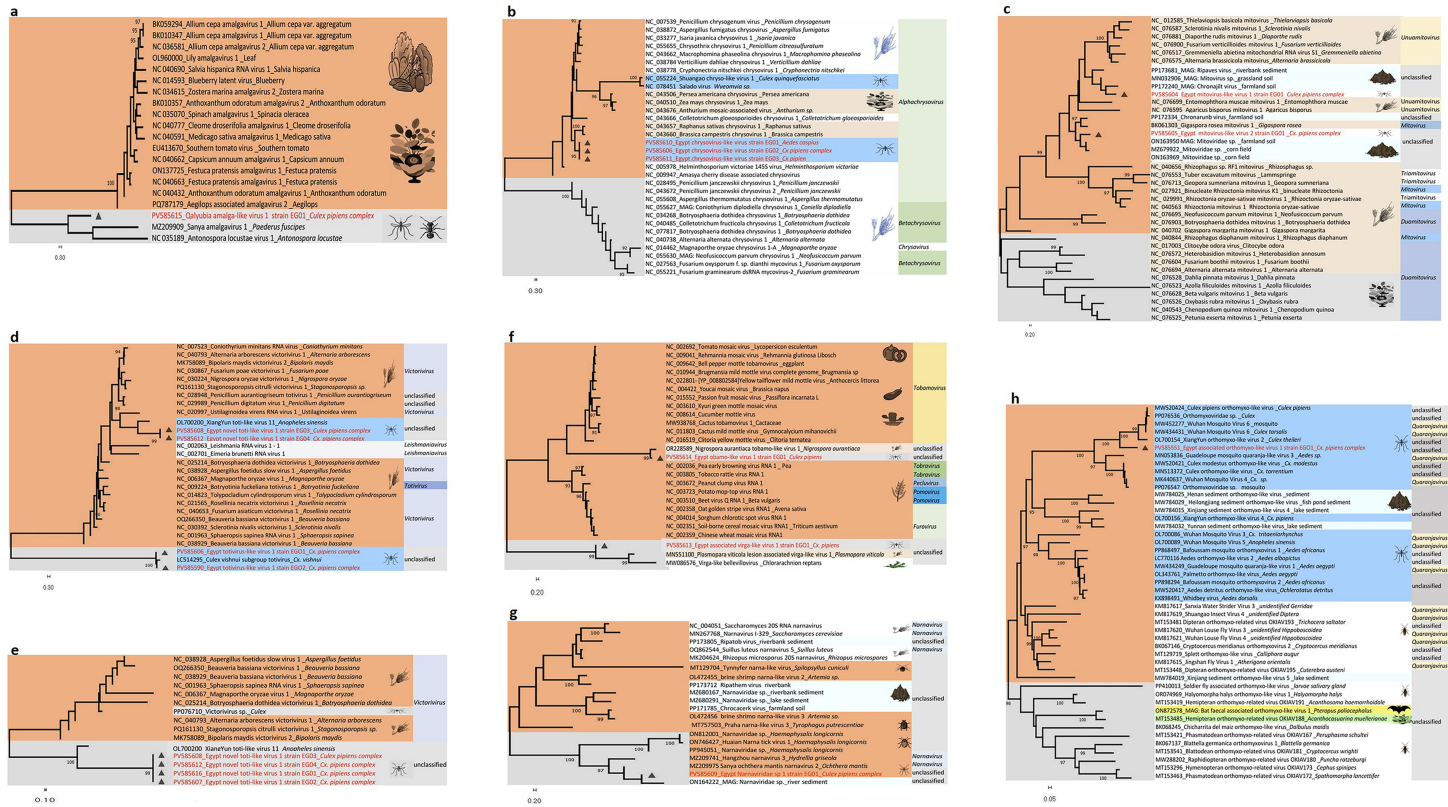
Phylogenetic analysis for amino acid (aa) of putative novel viruses from seven families identified by meta-viromic sequencing for mosquitoes in Egypt. (**a**) Phylogenetic tree of the RNA-dependent RNA polymerase (RdRp) aa sequences of *Amalgavirus* (Amalgaviridae). (**b**) Phylogenetic trees for aa sequence of RdRp of Chrysoviridae. (**c**) Phylogenetic trees for aa sequence of RdRp of Mitoviridae. (**d**) Phylogenetic trees for aa sequence of RdRp of Totiviridae. (**e**) Phylogenetic trees for aa sequence of capsid protein of Totiviridae. (**f**) Phylogenetic trees for aa sequence of replicase protein of Virgaviridae. (**g**) Phylogenetic trees for aa sequence of replicase protein of Narnaviridae. (**h**) Phylogenetic trees for aa sequence of replicase protein of Orthomyxoviridae. Bootstrap values (1,000 replicates, not shown for less than 90%) of neighbor-joining are shown above the main lineages. Triangles indicate sequences obtained in this study.

**Fig 4:**
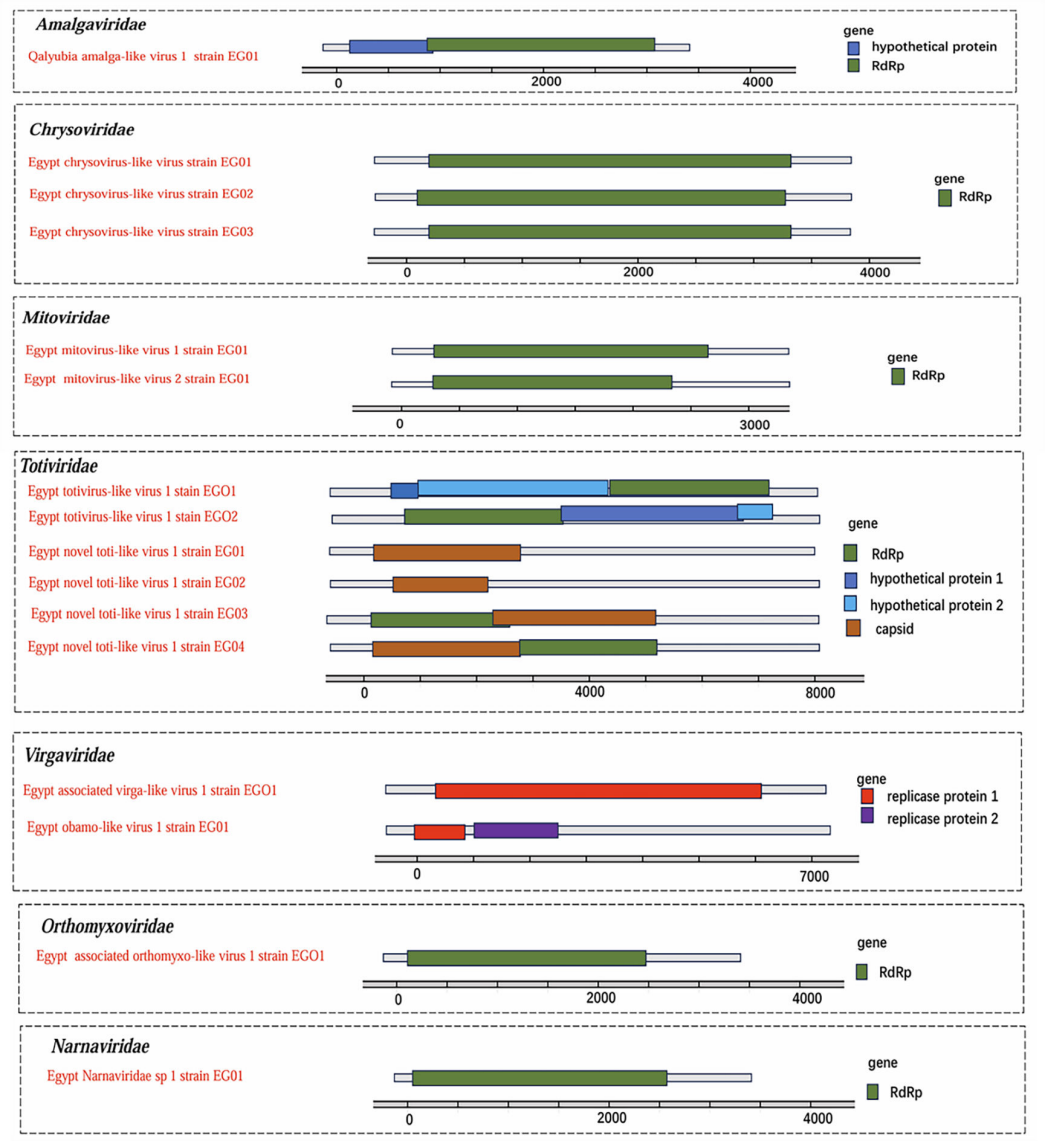
Genome organizations of 10 putative novel viruses identified from meta-viromic sequencing for mosquitoes in Egypt.

A total of 10 putative novel viruses were classified into seven viral families. Phylogenetic trees constructed based on amino acid sequences (Fig. 3) and genomic sequences (Fig. S5) reveal the phylogenetic relationships among 10 suspected novel viruses from seven viral families: Amalgaviridae, Chrysoviridae, Mitoviridae, Totiviridae, Virgaviridae, Narnaviridae, and Orthomyxoviridae. Figure 4 shows that genome organizations of 10 putative novel viruses identified from meta-viromic sequencing for mosquitoes in Egypt.

A putative novel plant RNA virus, designated Qalyubia amalga-like virus 1, was identified in the *Cx. pipiens* complex. This virus belongs to the genus *Amalgavirus* (family Amalgaviridae), whose members possess double-stranded and negative-strand RNA genomes (Fig. 3a). Another putative novel virus, Egypt chrysovirus-like virus, was assigned to the family Chrysoviridae based on the RdRp segments obtained from *Cx. pipiens* complex and *Ae. caspius* (Fig. 3b). Two putative novel viruses, namely Egypt mitovirus-like virus 1 and Egypt mitovirus-like virus 2, were classified within the family Mitoviridae (Fig. 3c). Additionally, two putative novel viruses—Egypt totivirus-like virus 1 and Egypt novel toti-like virus 1—belonged to the family Totiviridae (Fig. 3d and e). Two other putative novel viruses, Egypt-associated virga-like virus 1 and Egypt obamo-like virus 1, were identified as members of the family Virgaviridae (Fig. 3f). A putative novel virus, Egypt Narnaviridae sp. 1, was detected in the family Narnaviridae (Fig. 3g). Egypt- associated orthomyxo-like virus 1, a putative novel virus in the family Orthomyxoviridae (Fig. 3h), is noteworthy given that some members of this family can infect a broad range of hosts, including mammals (27). Table S2 provides the sequence similarity analysis of the amino acid (aa) sequences of these putative novel viruses from the seven families, identified through meta-viromic sequencing of mosquitoes in Egypt. The aforementioned genomic sequence data have been deposited in the NCBI GenBank database, with the corresponding accession numbers provided in Table S3.

### Mosquito virome differences among the GC, AS, and RS groups

**Fig 5:**
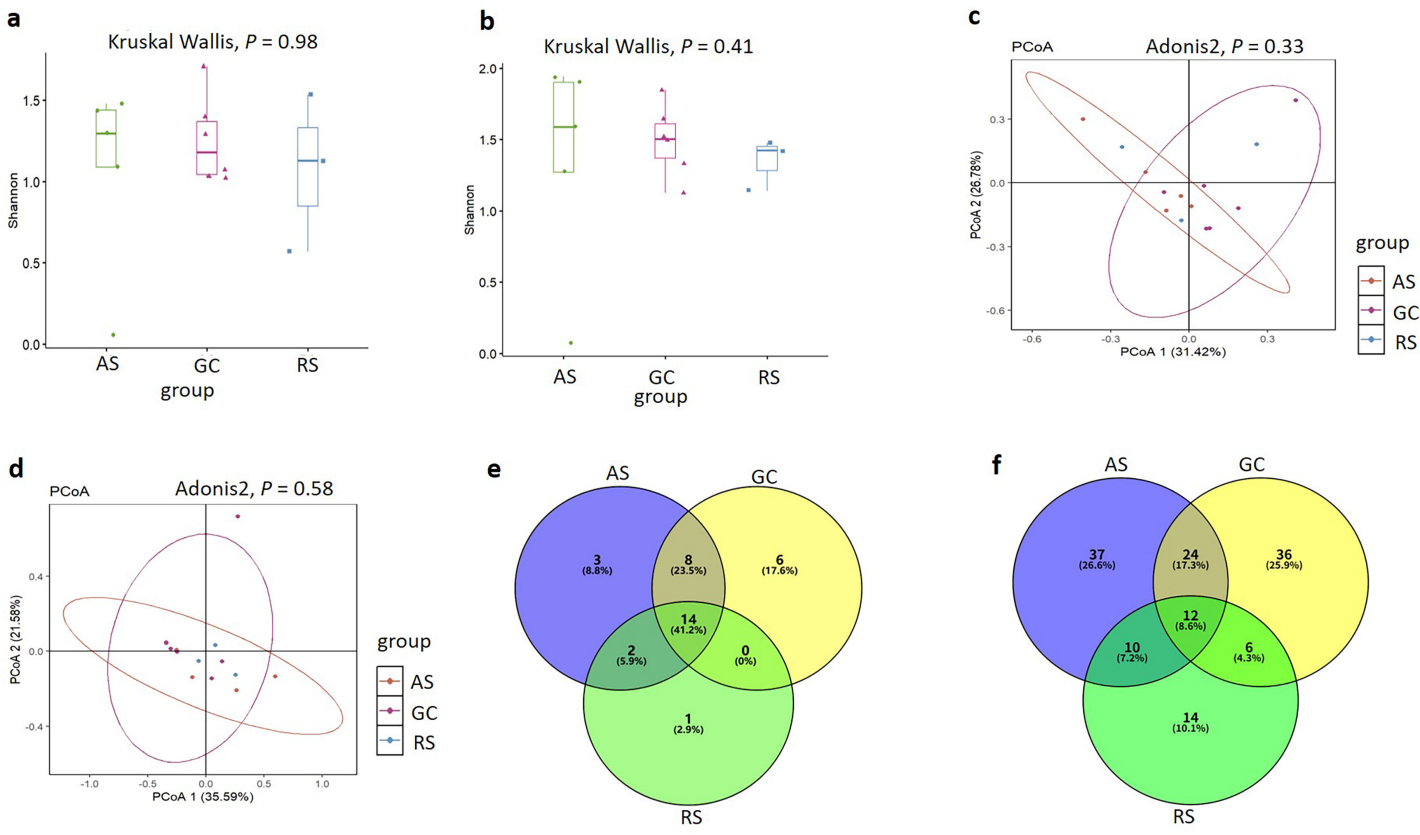
(**a**) Shannon diversity analysis of RNA viruses (family or equivalent taxonomic rank) among the AS, GC, and RS groups. (**b**) Shannon diversity analysis of RNA viruses (species or equivalent taxonomic rank) among the AS, GC, and RS groups. (**c**) Principal coordinate analysis (PCoA) of RNA virus compositions (family or equivalent taxonomic rank) among the AS, GC, and RS groups. (**d**) PCoA of RNA virus compositions (species or equivalent taxonomic rank) among the AS, GC, and RS groups. (**e**) Venn diagram showing the overlap of viruses identified at the family or equivalent rank. (**f**) Venn diagram showing the overlap of viruses at the species level. All statistical analyses were performed based on viral relative abundance (i.e., relative abundance percentage of RPKM). RPKM: reads per kilobase of transcript per million mapped reads. Group definitions: GC group (GC) comprises mosquito pools sampled from the Greater Cairo and Nile Valley-Delta regions (Cairo, Giza, Al Qalyubia, and Fayoum); AS group (AS) comprises mosquito pools sampled from the Nile Valley-Upper Egypt region (Aswan); RS group (RS) comprises mosquito pools sampled from the Red Sea region (Hurghada).

Shannon index analysis was conducted to evaluate the RNA virome composition at both the family and species levels, with statistical significance assessed using the Kruskal–Wallis test. The results demonstrated no statistically significant differences in virome diversity among the GC, AS, and RS groups (family level: *P* = 0.98; species level: *P* = 0.41) (Fig. 5a and b). Principal coordinate analysis (PCoA) based on Bray–Curtis dissimilarities, combined with PERMANOVA tests, was performed to compare the population distribution of the mosquito RNA virome. No significant variations in virome community structure were observed between the three groups at either the family (*P* = 0.33) or species level (*P* = 0.58) (Fig. 5c and d).

At the family level, 41.2% of the detected viruses were shared across all three groups (GC, AS, and RS) (Fig. 5e). In contrast, the overlap in viral species was substantially lower, with only 8.6% of viruses common to the GC, AS, and RS groups (Fig. 5f). Linear discriminant analysis effect size (LEfSe) analysis was employed to identify group-specific differential viruses at the species level (comparing AS, GC, and RS groups). Subsequently, subgroup differential analysis using the Wilcoxon test revealed that mosquitoes collected from Aswan (AS group) were significantly enriched in Diptera-related viruses (*P* < 0.05). However, LEfSe analysis at the family level (or equivalent taxonomic rank) failed to identify any significant differential marker viruses among the three groups.

## DISCUSSION

Meta-viromic sequencing enables the simultaneous characterization of the abundance and diversity of mosquito RNA viromes and is currently widely used to investigate RNA viromes across different geographical regions, host species, and climatic conditions (18, 28). In this study, we employed meta-viromic sequencing to characterize the RNA viromes of 14 mosquito pools, encompassing three species (*Cx. pipiens* complex, *Cx. perexiguus*, and *Ae*. caspius) collected from Egypt. We further profiled these RNA viromes at both the family and species taxonomic levels.

A total of over 130 virus species, belonging to 35 clusters (families or equivalent taxonomic ranks), were identified in the present study. Additionally, a substantial proportion of viruses in some pools could not be classified into established families. Notably, most of these unclassified viruses have been detected in arthropods via meta-viromic sequencing and are considered arthropod-specific (6). The most prevalent viral families identified in *Culex* mosquitoes from Egypt were Rhabdoviridae, Phasmaviridae, Iflaviridae, Narnaviridae, Flaviviridae, and Orthomyxoviridae. This viral family composition differs from the major viral families previously reported in a study of four common mosquito species from Shandong Province, China (29). Meanwhile, we identified multiple ISVs, including CxFV (Flaviviridae), Kustavi Toti-like virus, Hanko Totivirus 5 (Totiviridae), CPLV (Phasmaviridae), Iflavirus sp., Culex Iflavi-like virus 1, Culex Iflavi-like virus 4 (Iflaviridae), GCRV (Rhabdoviridae), and Sarawak virus (Alphatetraviridae), etc. Phylogenetic analysis of these ISVs revealed their closest evolutionary relationships with viral genome sequences from the Middle East, Europe, Oceania, and Asia. Furthermore, we detected several viruses (Wuhan house fly virus 1, Zhejiang mosquito virus 3, and Wuhan mosquito virus 6) that had previously been identified in mosquitoes from Asian countries (6, 10, 30). Collectively, these results indicate the co-circulation of multiple ISVs in Egyptian mosquitoes, as well as the global transmission potential and broad host range of these viruses.

Egypt is a typical tropical country, featuring a tropical Mediterranean climate along its northern coast and a tropical desert climate across the remainder of its territory. This study significantly expands the known spectra of viruses associated with *Culex* and *Aedes* mosquitoes under Egyptian climatic conditions. To the best of our knowledge, this represents the first in-depth investigation of mosquito RNA viromes in Egypt, covering regions including the Greater Cairo and Nile Valley-Delta, the Nile Valley-Upper Egypt, and the Red Sea Governorate. The mosquito sampling sites spanned diverse regions of Egypt, encompassing both tropical Mediterranean and tropical desert climates. The objectives of this study were to characterize the RNA viromes of three common mosquito species (*Cx. pipiens* complex, *Cx. perexiguus*, and *Ae. caspius*), clarify the diversity of associated viruses, and determine the potential vector roles of these mosquito species. Analysis of the mosquito viromes revealed differences in the viral species carried by *Culex* mosquitoes across different regions although these differences did not reach statistical significance. Less than 10% of the detected viruses were shared among all *Culex* mosquitoes across the sampled areas; specifically, only 8.6% of viruses were common to the mosquito viromes of the three areas.

Previous studies have indicated that mosquitoes infected with ISVs may display increased susceptibility to pathogenic flaviviruses (31, 32). CxFV as a well-characterized ISV can be categorized into two distinct genotypes: the USA/Asia genotype (Clade 1) and the Latin American/Caribbean/Africa genotype (Clade 2) (33). First isolated from *Cx. pipiens* in Japan in 2007 (34), CxFV has since been detected in multiple mosquito species—including *Cx. quinquefasciatus*, *Cx. pipiens*, *Cx. tritaeniorhynchus*, *Cx. restuans*, and *Anopheles sinensis*—across diverse geographical regions such as China, the USA, Japan, Mexico, Uganda, and Brazil (35–39). In the present study, mosquito sampling sites in Egypt covered areas with both tropical Mediterranean and tropical desert climates. Phylogenetic analysis revealed that CxFV strains identified from Egyptian mosquitoes are more closely related to the tropical lineage and Clade 2. Notably, CxFV was detected in both the *Cx. pipiens* complex and *Aedes caspius* in Egypt; a particularly high prevalence of CxFV was observed in the *Cx. pipiens* complex from Aswan, suggesting the widespread distribution of this virus in the region. Cytological experiments have confirmed that CxFV can induce cell-fusion cytopathic effects in C6/36 cells (38, 40, 41). Although CxFV infection is currently thought to be restricted to mosquitoes, increased prevalence of mosquito-borne flaviviruses may trigger cytopathic effects in vertebrate cells when co-occurring with other human pathogens (42). Importantly, the presence of CxFV implies an elevated risk of infection with zoonotic mosquito-borne viruses, such as West Nile virus (43, 44). We also identified another ISV, Sarawak virus, in Egyptian mosquitoes. Sarawak virus (strain SWK-M26) was first isolated from *Ae. albopictus* in Malaysia; it exclusively induces cytopathic lesions in the C6/36 cell line (45) and is classified as an ISV. The genome sequence of Sarawak virus obtained in this study exhibited a high degree of similarity to Shinobi tetravirus from Japan—a virus previously shown to inhibit flavivirus replication (46). Collectively, these findings underscore the significance of leveraging high-throughput sequencing data for mosquito vector surveillance in Egypt, as such approaches enable the comprehensive identification of ISVs and assessment of their potential implications for public health.

In this study, we conducted a comprehensive survey of mosquito viromes across most regions of Egypt. The results confirm both the diversity of potential viral hosts and the high viral diversity associated with three mosquito species—Cx. pipiens complex, *Cx. perexiguus*, and *Aedes caspius*—in Egypt. Notably, we identified a putative novel virus, Qaluobiyah amalga-like virus 1, belonging to the family Amalgaviridae, from the virome of the *Cx. pipien*s complex. Phylogenetic analysis showed that this virus clusters with other amalgaviruses detected in arthropods (e.g., *Paederus fuscipes*). This is notable because previously identified members of the Amalgaviridae family have primarily been plant viruses (47). The family Virgaviridae is known to consist exclusively of plant viruses (48). However, two putative novel viruses identified in our study—Egypt-associated Virga-like virus 1 and Egypt Obamo-like virus 1—clustered with viral sequences derived from fungi and the marine alga *Chlorarachnion reptans*, expanding our understanding of the host range of Virgaviridae-related viruses. Phylogenetic analysis of Narnaviridae sequences from our study suggests that arthropods—including *Cx. pipiens* complex mosquitoes, *Ochthera mantis* (predatory mantis fly or shore fly), and *Haemaphysalis longicornis* (Asian longhorned or cattle tick)—may serve as potential hosts for these viruses, which broadens the known host spectrum of Narnaviridae. For other viral families, chrysoviruses are known to infect fungi, plants, and insects and may induce hypovirulence in their fungal hosts (49). Mitoviruses (family Mitoviridae) have a broad distribution across fungi, plants, and invertebrates (50). The family Totiviridae traditionally infects lower eukaryotes such as filamentous fungi, yeasts, and protozoa, but its host range has recently expanded to include insects, plants, fish, crustaceans, and bats (51). It is particularly noteworthy that we identified a putative novel virus, Egypt-associated Orthomyxo-like virus 1, belonging to the family Orthomyxoviridae. This family includes four influenza virus genera (Alpha-, Beta-, Delta-, and Gammainfluenzavirus), as well as Thogoto virus, Isa virus, and Quaranja virus (27). Orthomyxoviridae viruses are known to cause influenza in vertebrates, including birds, humans, and other mammals (27, 52), making the expansion of our knowledge regarding this viral family clinically and epidemiologically significant. In conclusion, our study identified 10 putative novel viruses belonging to seven viral families. Additionally, a large proportion of unclassified viruses detected were determined to be arthropod-specific. By filling a critical gap in the characterization of mosquito viromes in Egypt, this study represents a key advancement in our understanding of viral diversity in this region.

Nevertheless, this study has several limitations that should be acknowledged. First, to investigate the RNA viromes carried by vector mosquitoes in Egypt, we collected a relatively small number of mosquito samples, which were restricted to six governorates: Cairo, Giza, Qalyubia, Faiyum, Aswan, and the Red Sea Governorate. Second, although Egypt is home to multiple mosquito species capable of harboring RNA viruses, our analysis was limited to three species: *Ae. caspius*, Culex *Cx. perexiguus*, and *Cx. pipiens* complex. Third, this study utilized metagenomic sequencing at the pooled sample level, where individual pools contained mosquitoes varying in both size and quantity. This pooling strategy may introduce bias, as the results could be disproportionately influenced by a small number of individual mosquitoes carrying high titers of specific virus families or species. However, this approach may also partially offset the constraints imposed by the limited overall sample size. Finally, this study did not finish the isolation of arboviruses or related cell culture infection experiments, which somewhat limits its direct contributions to understanding infectious diseases in Egypt. Looking forward, we aim to conduct in-depth research on arboviruses in Egypt, with a focus on exploring the replication and transmission dynamics of these viruses in the region at a more mechanistic level.

### Conclusion

Through total RNA sequencing of 654 mosquitoes collected in Egypt, we identified more than 130 viruses spanning 35 taxonomic clusters (families or equivalent ranks). A comparative analysis of the viromes from three vector species—*Ae*. *caspius*, *Cx. perexiguus*, and the *Cx. pipiens* complex—revealed that despite their overlapping geographic distributions, the RNA virome compositions (at both the family and species levels) differed moderately among these species. Additional variations were also observed across geographic locations within the current sampling scope. In this study, we identified multiple ISVs, including CxFV, Kustavi Toti-like virus, Hanko Totivirus 5, CPLV, Culex Iflavi-like virus 1, Culex Iflavi-like virus 4, GCRV, and Sarawak virus, etc. Phylogenetic analysis indicated that these ISVs share the closest evolutionary relationships with viral genome sequences from the Middle East, Europe, Oceania, and Asia. Notably, in samples of the *Cx. pipiens* complex, a large proportion of viral contigs were assigned to the families Rhabdoviridae, Flaviviridae, Iflaviridae, and Phasmaviridae. Species-level analysis further confirmed that these contigs primarily corresponded to GCRV, CxFV, and CPLV. Collectively, these findings demonstrate the co-circulation of multiple ISVs in mosquitoes from Egypt. In conclusion, this study has significantly expanded our knowledge of both known and previously uncharacterized mosquito-associated viruses in Egypt. These results also highlight the necessity of conducting more in-depth investigations into arthropod viromes (encompassing insects and ticks) within the country. Looking ahead, we anticipate more detailed studies to elucidate the complex relationships between mosquitoes, viruses, host species, and the environment in Egypt and other African countries. Such research will ultimately facilitate a comprehensive understanding of viral transmission dynamics and may lead to the development of novel biocontrol tools targeting disease-transmitting mosquito vectors—with applications in Egypt and worldwide.

## Data Availability

The sequencing Raw data and nucleotide sequences produced in this study have been deposited in the NCBI Sequence Read Archive (SRA) database under the BioProject accession number PRJNA1235869 and GenBank PV585540–PV585616.

## SUPPLEMENTAL MATERIAL

Fig. S1 spectrum.02135-25-s0001.pdf Map of Egypt (survey sites are shown on map).

Fig. S2 spectrum.02135-25-s0002.pdf RNA viral profiles detected by meta-viromic sequencing (family level).

Fig. S3 spectrum.02135-25-s0003.pdf Venn diagrams illustrate the overlap of viruses carried by different mosquito species, at the family (or equivalent taxonomic rank) (a) and species levels (b).

Fig. S4 spectrum.02135-25-s0004.pdf Phylogenetic analysis for *Culex flavivirus* (Flaviviridae) partial non-structural 5 gene.

Fig. S5 spectrum.02135-25-s0005.pdf Phylogenetic analysis for ten putative novel viruses with 16 genome sequences from seven families identified by meta-viromic sequencing for mosquitoes in Egypt.

Supplemental material spectrum.02135-25-s0006.docx Supplemental figure legends.

Table S1 spectrum.02135-25-s0007.xlsx Summary of different mosquito pools, captured in Egypt.

Table S2 spectrum.02135-25-s0008.docx Sequence similarity analysis of putative novel viruses' aa sequence from seven families identified by meta-viromic sequencing for mosquitoes in Egypt.

Table S3 spectrum.02135-25-s0009.xlsx The family (or equivalent taxonomic rank) information and GenBank ID of the genome sequences generated in this study.

ASM does not own the copyrights to Supplemental Material that may be linked to, or accessed through, an article. The authors have granted ASM a non-exclusive, world-wide license to publish the Supplemental Material files. Please contact the corresponding author directly for reuse.
