# Supplementary material for: Metagenomic sequencing reveals viral diversity of mosquitoes from Egypt: co-circulation of multiple insect-specific viruses
Source: Microbiol Spectr. 2026 Mar 6;14(4):e02135-25. doi: 10.1128/spectrum.02135-25 (PMC13055307; doi:10.1128/spectrum.02135-25)
Supplement: Fig. S3 — Venn diagrams illustrate the overlap of viruses carried by different mosquito species, at the family (or equivalent taxonomic rank) (a) and species levels (b). [file spectrum.02135-25-s0003.pdf]

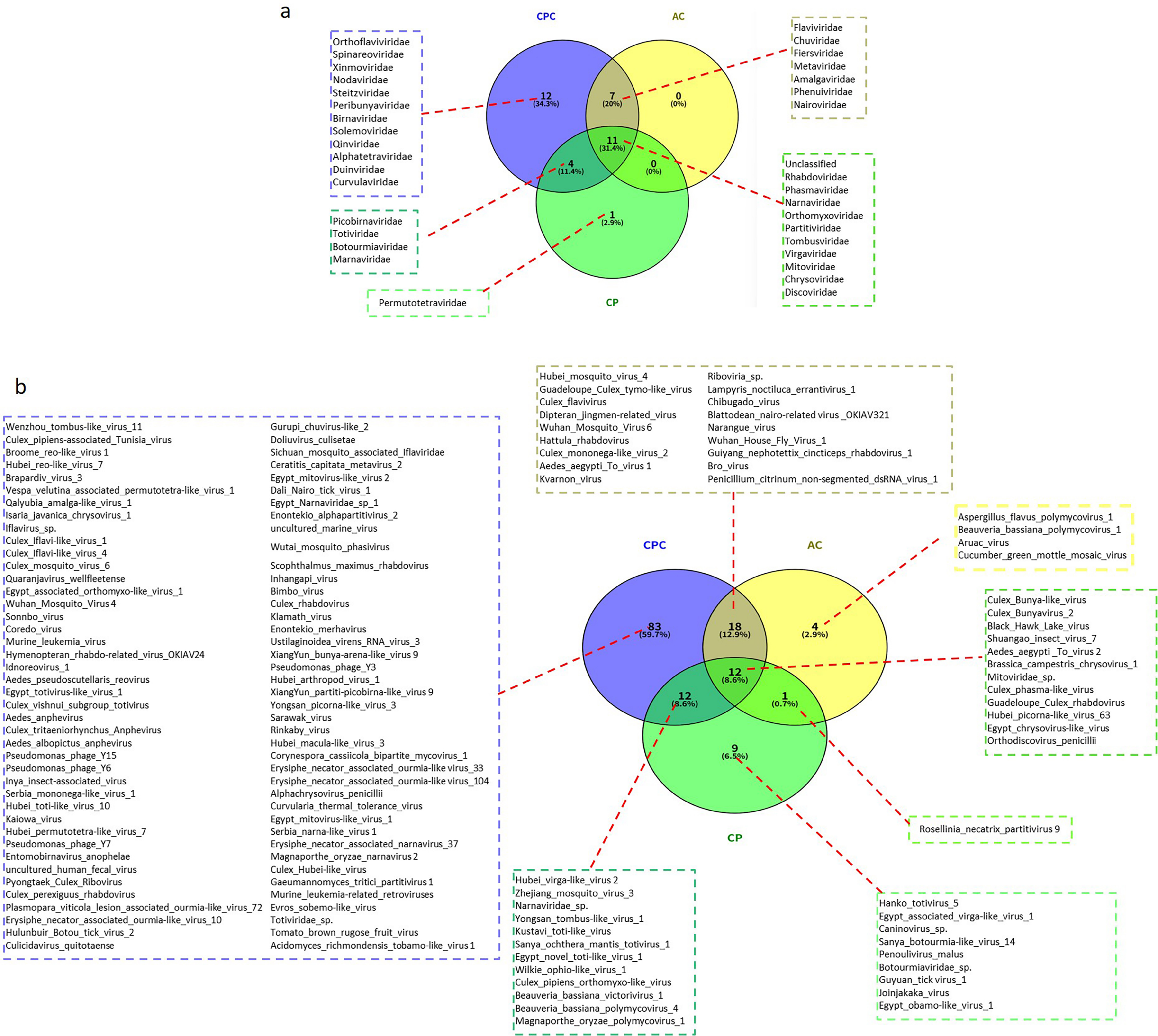
a
CPC
AC
CP
12 (34.3%)
7 (20%)
0 (0%)
11 (31.4%)
4 (11.4%)
0 (0%)
1 (2.9%)
Orthoflaviviridae
Spinareoviridae
Xinmoviridae
Nodaviridae
Steitzviridae
Peribunyaviridae
Birnaviridae
Solemoviridae
Qinviridae
Alphatetraviridae
Duinviridae
Curvulaviridae
Picobirnaviridae
Totiviridae
Botourmiaviridae
Marnaviridae
Permutotetraviridae
Flaviviridae
Chuviridae
Fiersviridae
Metaviridae
Amalgaviridae
Phenuiviridae
Nairoviridae
Unclassified
Rhabdoviridae
Phasmaviridae
Narnaviridae
Orthomyxoviridae
Partitiviridae
Tombusviridae
Virgaviridae
Mitoviridae
Chrysoviridae
Discoviridae
b
CPC
AC
CP
83 (59.7%)
18 (12.9%)
4 (2.9%)
12 (8.6%)
12 (8.6%)
1 (0.7%)
9 (6.5%)
Wenzhou_tombus-like_virus_11
Culex_pipiens-associated_Tunisia_virus
Broome_reo-like_virus 1
Hubei_reo-like_virus_7
Brapardiv_virus_3
Vespa_velutina_associated_permutotetra-like_virus_1
Qalyubia_amalga-like_virus_1
Isaria_javanica_chrysovirus_1
Iflavirus_sp.
Culex_Iflavi-like_virus_1
Culex_Iflavi-like_virus_4
Culex_mosquito_virus_6
Quaranjavirus_wellfleetense
Egypt_associated_orthomyxo-like_virus_1
Wuhan_Mosquito_Virus 4
Sonnbo_virus
Coredo_virus
Murine_leukemia_virus
Hymenopteran_rhabdo-related_virus_OKIAV24
Idnoreovirus_1
Aedes_pseudoscutellaris_reovirus
Egypt_totivirus-like_virus_1
Culex_vishnui_subgroup_totivirus
Aedes_anphevirus
Culex_tritaeniorhynchus_Anphevirus
Aedes_albopictus_anphevirus
Pseudomonas_phage_Y15
Pseudomonas_phage_Y6
Inya_insect-associated_virus
Serbia_mononega-like_virus_1
Hubei_toti-like_virus_10
Kaiowa_virus
Hubei_permutotetra-like_virus_7
Pseudomonas_phage_Y7
Entomobirnavirus_anophelae
uncultured_human_fecal_virus
Pyongtaek_Culex_Ribovirus
Culex_perexiguus_rhabdovirus
Plasmopara_viticola_lesion_associated_ourmia-like_virus_72
Erysiphe_necator_associated_ourmia-like_virus_10
Hulunbuir_Botou_tick_virus_2
Culicidavirus_quitotaense
Gurupi_chuvirus-like_2
Doliuvirus_culisetae
Sichuan_mosquito_associated_Iflaviridae
Ceratitis_capitata_metavirus_2
Egypt_mitovirus-like_virus 2
Dali_Nairo_tick_virus_1
Egypt_Narnaviridae_sp_1
Enontekio_alphapartitivirus_2
uncultured_marine_virus
Wutai_mosquito_phasivirus
Scophthalmus_maximus_rhabdovirus
Inhangapi_virus
Bimbo_virus
Culex_rhabdovirus
Klamath_virus
Enontekio_merhavirus
Ustilaginoidea_virens_RNA_virus_3
XiangYun_bunya-arena-like_virus 9
Pseudomonas_phage_Y3
Hubei_arthropod_virus_1
XiangYun_partiti-picobirna-like_virus 9
Yongsan_picorna-like_virus_3
Sarawak_virus
Rinkaby_virus
Hubei_macula-like_virus_3
Corynespora_cassiicola_bipartite_mycovirus_1
Erysiphe_necator_associated_ourmia-like virus_33
Erysiphe_necator_associated_ourmia-like virus_104
Alphachrysovirus_penicillii
Curvularia_thermal_tolerance_virus
Egypt_mitovirus-like_virus_1
Serbia_narna-like_virus 1
Erysiphe_necator_associated_narnavirus_37
Magnaporthe_oryzae_narnavirus 2
Culex_Hubei-like_virus
Gaeumannomyces_tritici_partitivirus 1
Murine_leukemia-related_retroviruses
Evros_sobemo-like_virus
Totiviridae_sp.
Tomato_brown_rugose_fruit_virus
Acidomyces_richmondensis_tobamo-like_virus 1
Hubei_mosquito_virus_4
Guadeloupe_Culex_tymo-like_virus
Culex_flavivirus
Dipteran_jingmen-related_virus
Wuhan_Mosquito_Virus 6
Hattula_rhabdovirus
Culex_mononega-like_virus_2
Aedes_aegypti_To_virus 1
Kvarnon_virus
Riboviria_sp.
Lampyris_noctiluca_errantivirus_1
Chibugado_virus
Blattodean_nairo-related virus _OKIAV321
Narangue_virus
Wuhan_House_Fly_Virus_1
Guiyang_nephotettix_cincticeps_rhabdovirus_1
Bro_virus
Penicillium_citrinum_non-segmented_dsRNA_virus_1
Aspergillus_flavus_polymycovirus_1
Beauveria_bassiana_polymycovirus_1
Aruac_virus
Cucumber_green_mottle_mosaic_virus
Culex_Bunya-like_virus
Culex_Bunyavirus_2
Black_Hawk_Lake_virus
Shuangao_insect_virus_7
Aedes_aegypti _To_virus 2
Brassica_campestris_chrysovirus_1
Mitoviridae_sp.
Culex_phasma-like_virus
Guadeloupe_Culex_rhabdovirus
Hubei_picorna-like_virus_63
Egypt_chrysovirus-like_virus
Orthodiscovirus_penicillii
Rosellinia_necatrix_partitivirus 9
Hubei_virga-like_virus 2
Zhejiang_mosquito_virus_3
Narnaviridae_sp.
Yongsan_tombus-like_virus_1
Kustavi_toti-like_virus
Sanya_ochthera_mantis_totivirus_1
Egypt_novel_toti-like_virus_1
Wilkie_ophio-like_virus_1
Culex_pipiens_orthomyxo-like_virus
Beauveria_bassiana_victorivirus_1
Beauveria_bassiana_polymycovirus_4
Magnaporthe_oryzae_polymycovirus_1
Hanko_totivirus_5
Egypt_associated_virga-like_virus_1
Caninovirus_sp.
Sanya_botourmia-like_virus_14
Penoulivirus_malus
Botourmiaviridae_sp.
Guyuan_tick virus_1
Joinjakaka_virus
Egypt_obamo-like_virus_1
