# Supplementary figures and images for: Metagenomic sequencing reveals viral diversity of mosquitoes from Egypt: co-circulation of multiple insect-specific viruses
Source: Microbiol Spectr. 2026 Mar 6;14(4):e02135-25. doi: 10.1128/spectrum.02135-25 (PMC13055307; doi:10.1128/spectrum.02135-25)

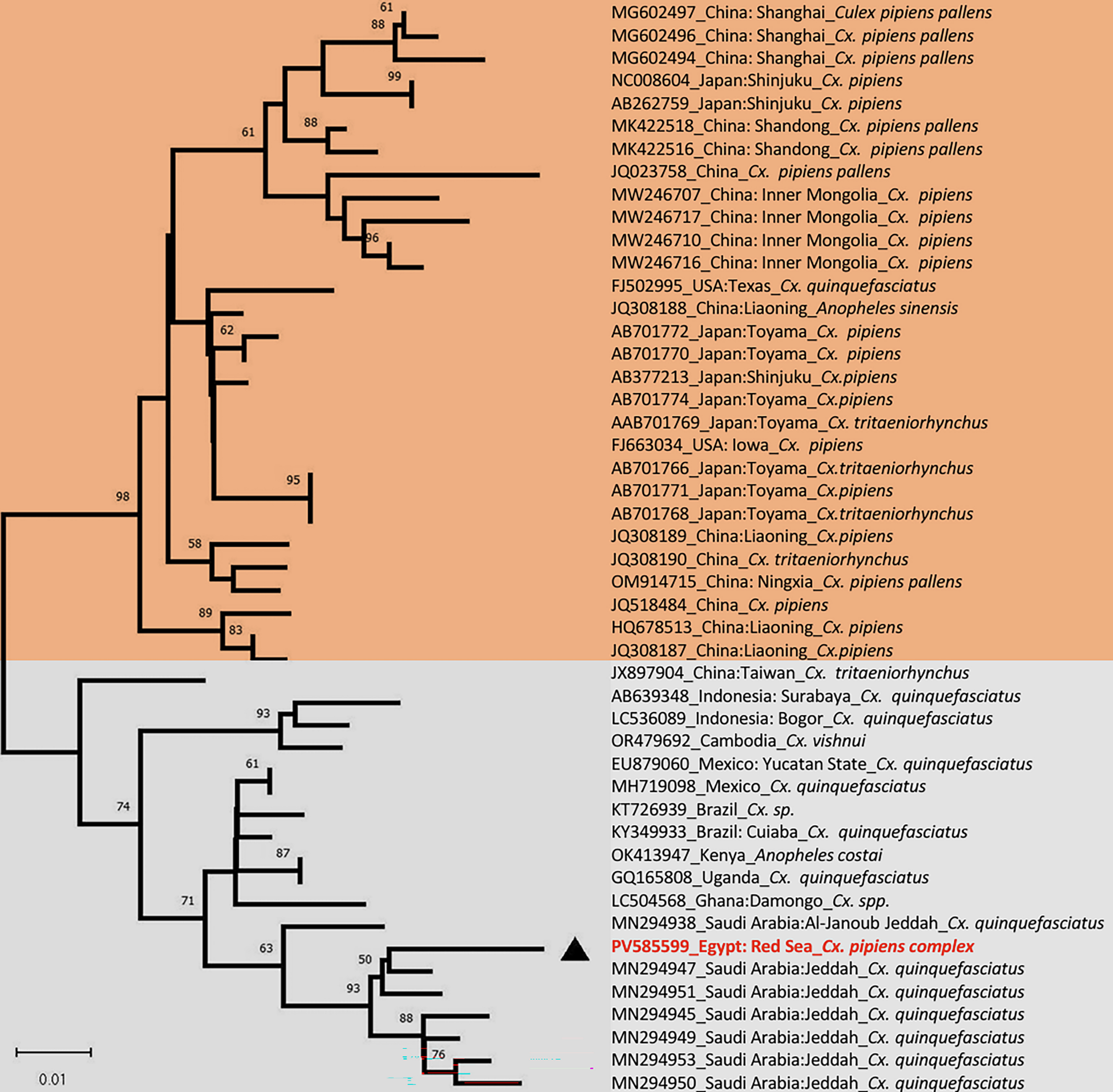

Supplement: Fig. S4 — Phylogenetic analysis for Culex flavivirus (Flaviviridae) partial non-structural 5 gene. [file spectrum.02135-25-s0004.pdf]

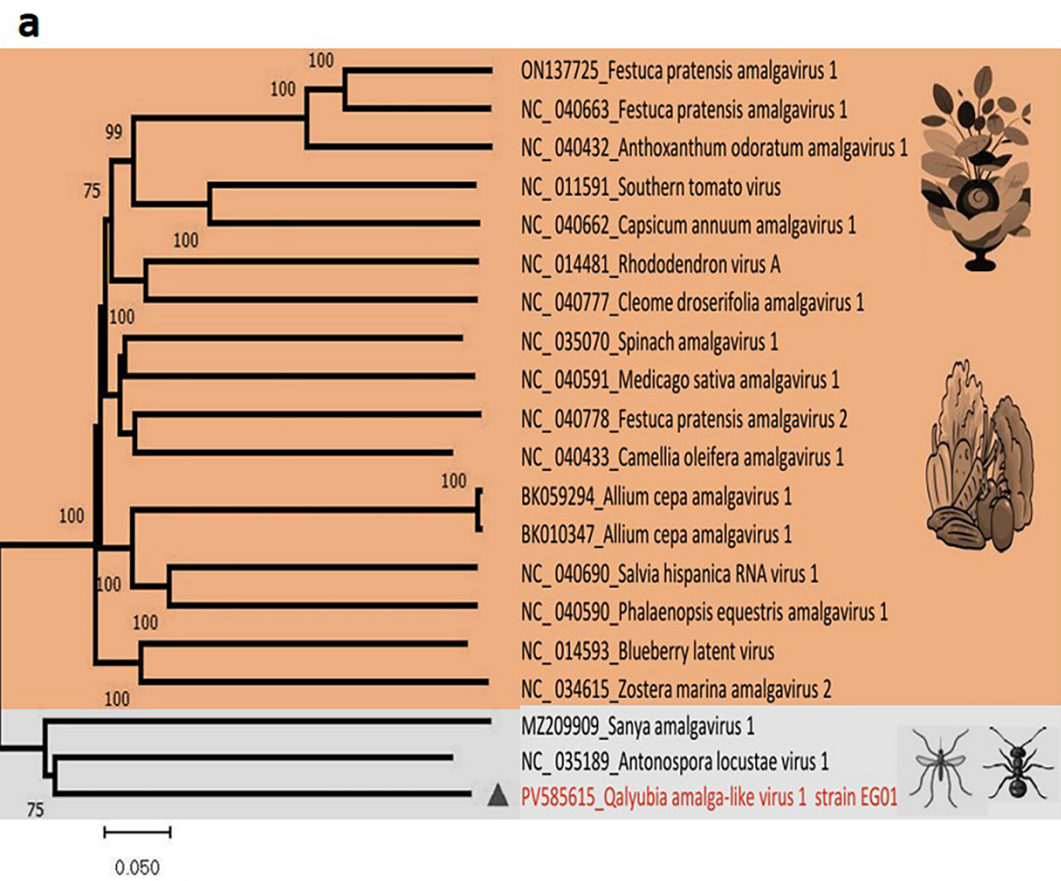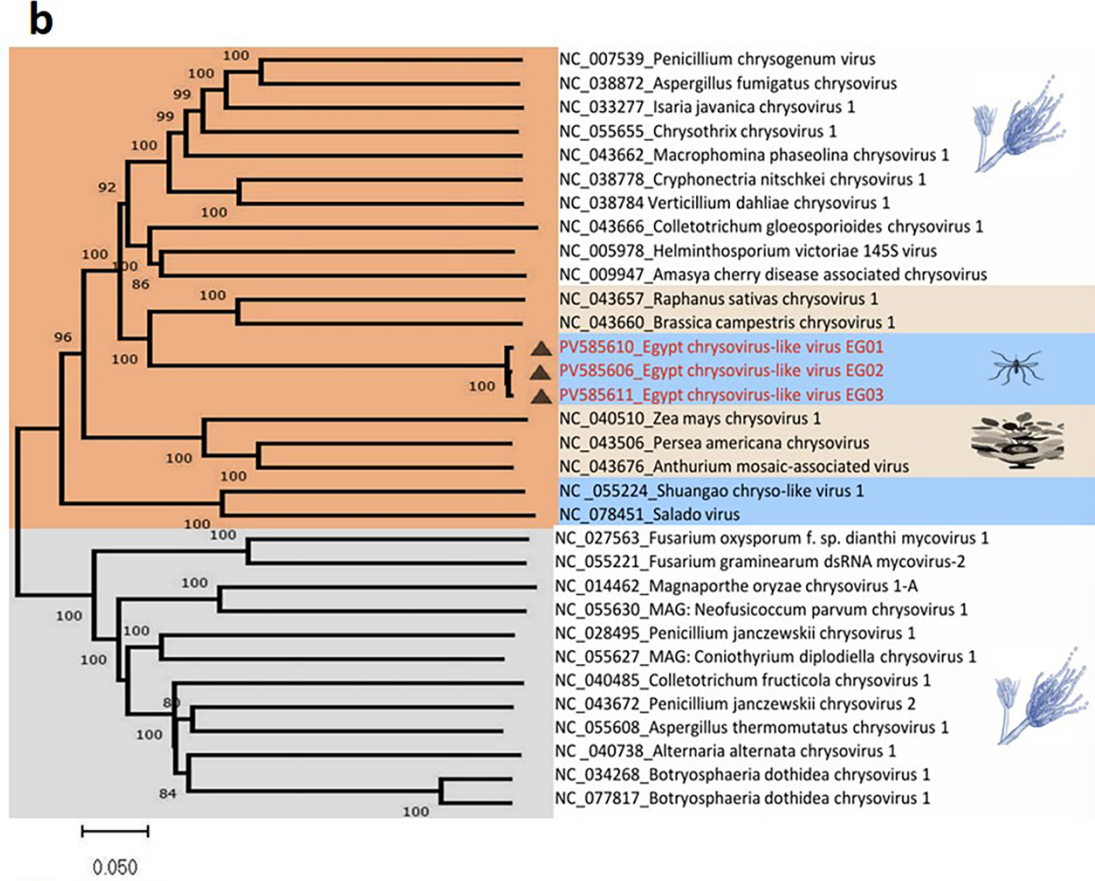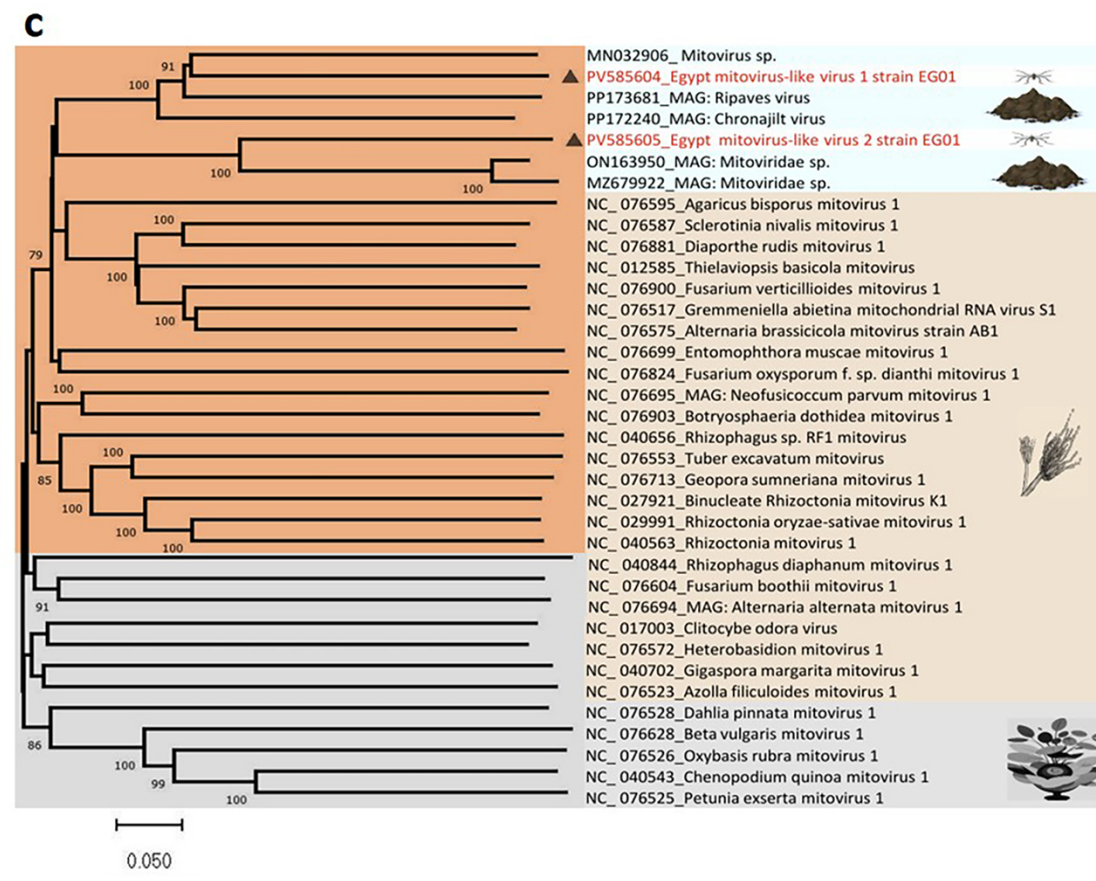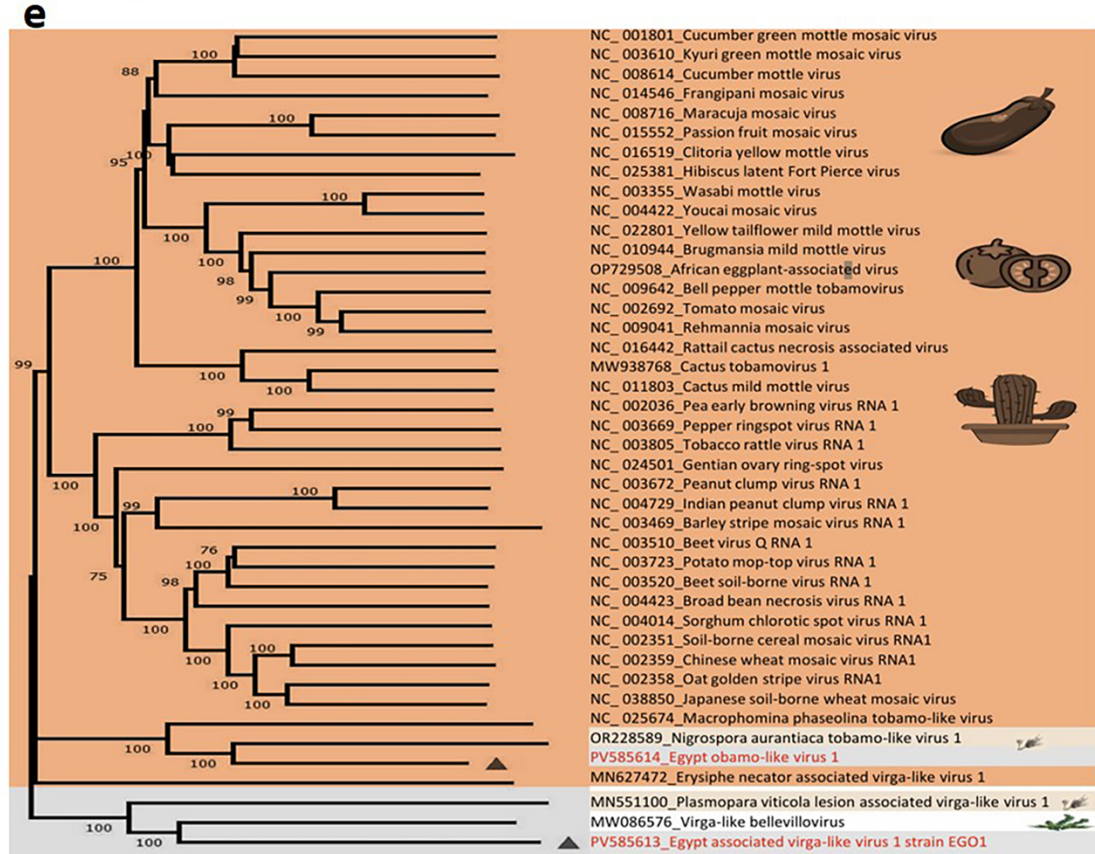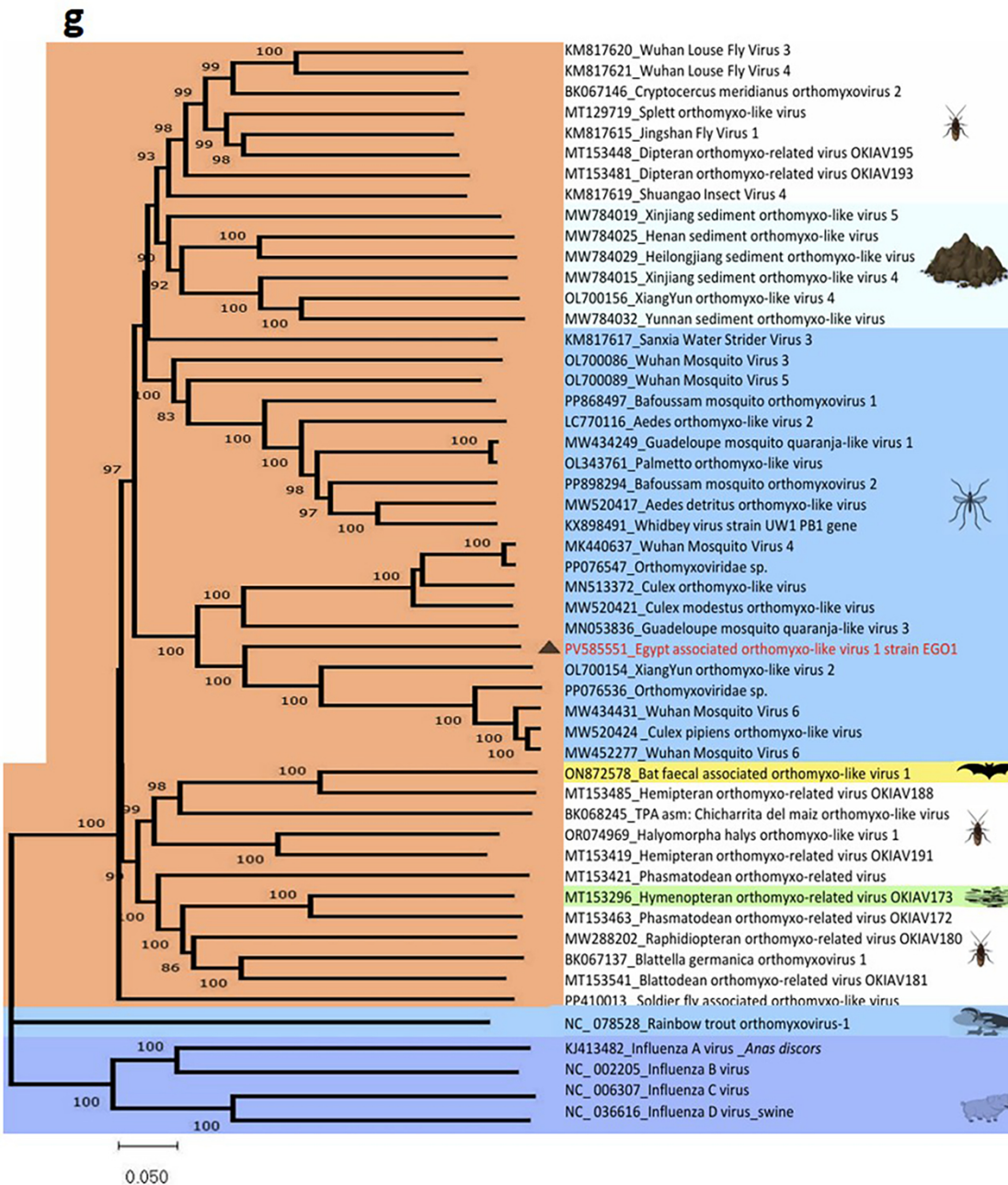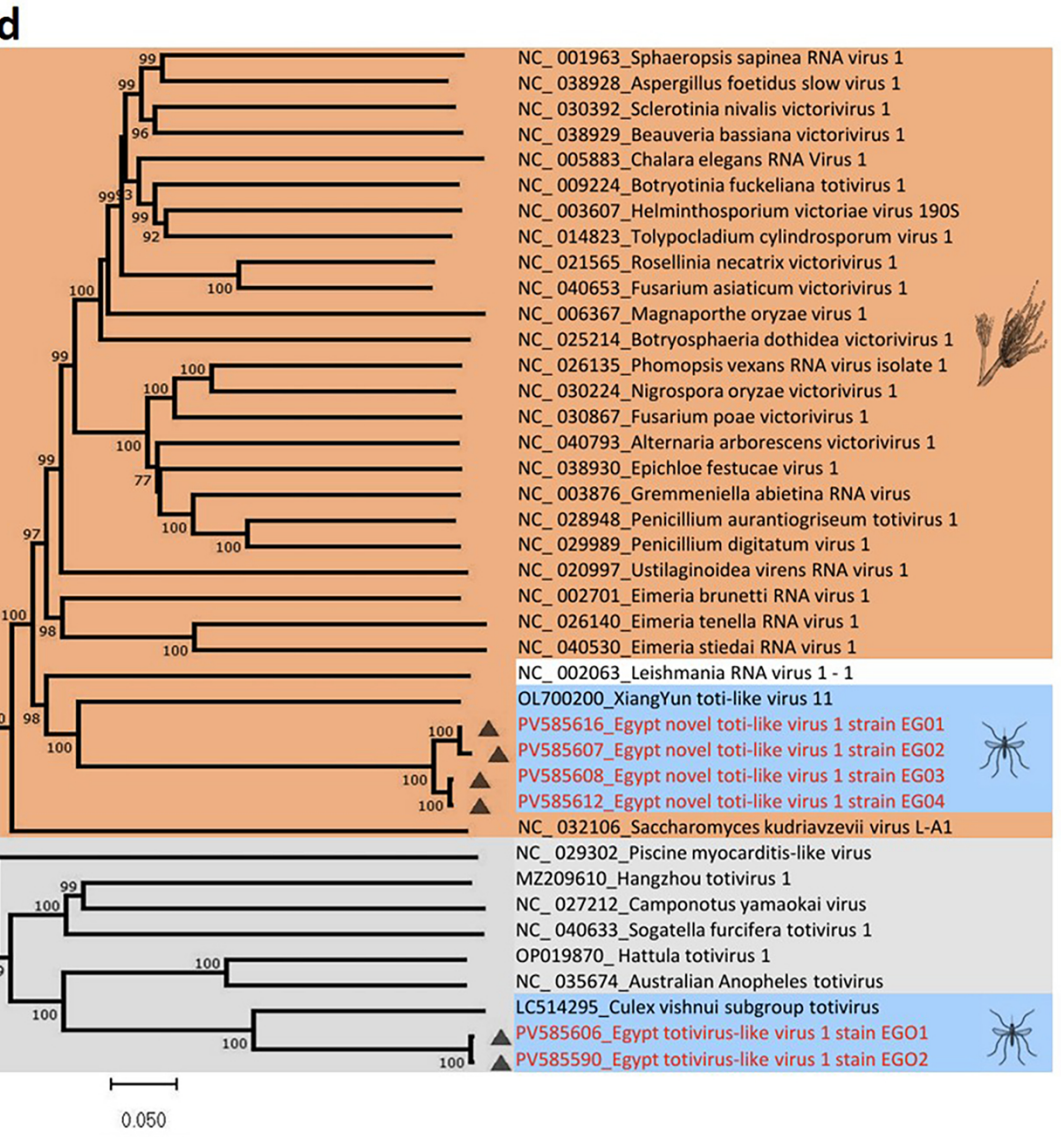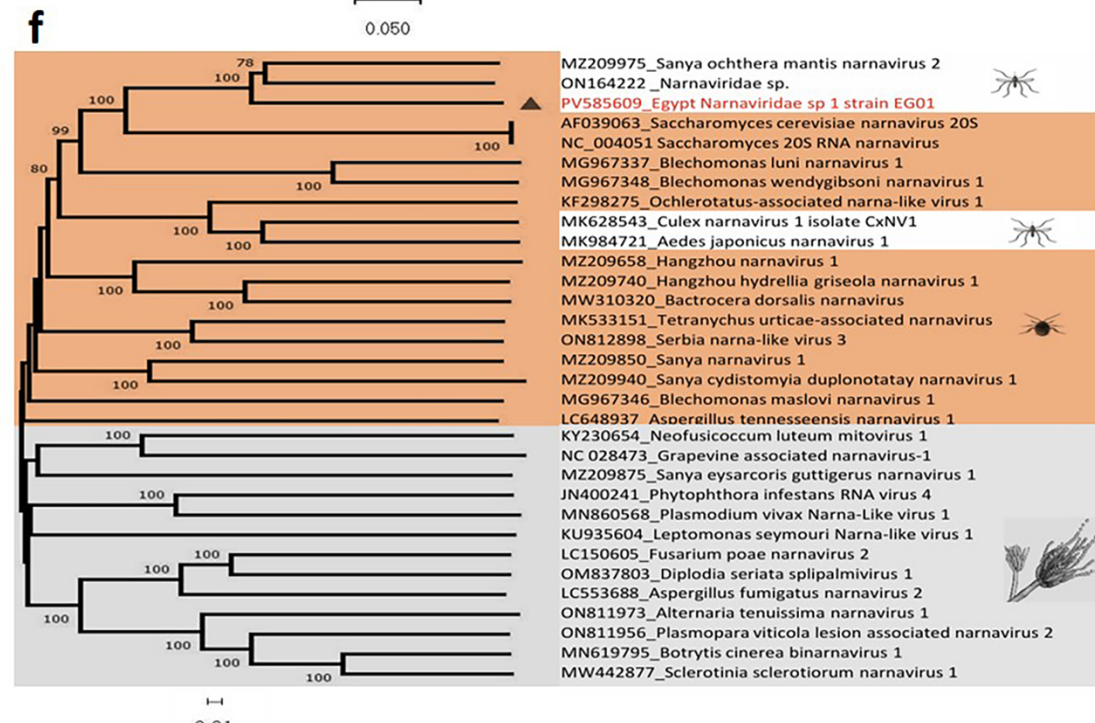

Supplement: Fig. S5 — Phylogenetic analysis for ten putative novel viruses with 16 genome sequences from seven families identified by meta-viromic sequencing for mosquitoes in Egypt. [file spectrum.02135-25-s0005.pdf]
