# Supplementary material for: Metagenomic sequencing reveals viral diversity of mosquitoes from Egypt: co-circulation of multiple insect-specific viruses
Source: Microbiol Spectr. 2026 Mar 6;14(4):e02135-25. doi: 10.1128/spectrum.02135-25 (PMC13055307; doi:10.1128/spectrum.02135-25)
Supplement: Supplemental material — Supplemental figure legends. [file spectrum.02135-25-s0006.docx]

**Supplementary Figure legends**

**Figure S1.** Map of Egypt (Survey sites are shown on Map). Sampling sites corresponding to the GC group are marked with red triangles, those in the AS group with yellow four-pointed stars, and those in the RS group with blue squares.

**Figure S2.** (a) RNA viral profiles detected by meta-viromic sequencing (family level). The heat map is based on the percentage of relative abundance of RPKM. RPKM: reads per kilobase of transcript per million mapped reads. CPC: *Culex pipiens complex*; AC: *Aedes caspius*; CP: *Culex perexiguus*.

**Figure S3.** Venn diagrams illustrate the overlap of viruses carried by different mosquito species, at the family (or equivalent taxonomic rank) (a) and species levels (b). Abbreviations: CPC: *Culex pipiens complex*; AC: *Aedes caspius*; CP: *Culex perexiguus*.

**Figure S4.** Phylogenetic analysis for Culex flavivirus (*Flaviviridae*) partial non-structural 5 gene. Bootstrap values (1000 replicates, not shown for less than 75% of neighbour-joining are shown above the main lineages, and the black triangles indicate sequences obtained in this study.

**Figure S5.** Phylogenetic analysis for ten putative novel viruses with 16 genome sequences from seven families identified by meta-viromic sequencing for mosquitoes in Egypt. (a) Phylogenetic trees for genomic sequences of Amalgaviridae. (b) Phylogenetic trees for genomic sequences of Chrysoviridae. (c) Phylogenetic trees for genomic sequences of Mitoviridae. (d) Phylogenetic trees for genomic sequences of Totiviridae. (e) Phylogenetic trees for genomic sequences of Virgaviridae. (f) Phylogenetic trees for genomic sequences of Narnaviridae. (g) Phylogenetic trees for genomic sequences of Orthomyxoviridae. Bootstrap values (1000 replicates, not shown for less than 75%) of neighbour-joining are shown above the main lineages. Triangles indicate sequences obtained in this study.
