# Supplementary material for: Metagenomic sequencing reveals viral diversity of mosquitoes from Egypt: co-circulation of multiple insect-specific viruses
Source: Microbiol Spectr. 2026 Mar 6;14(4):e02135-25. doi: 10.1128/spectrum.02135-25 (PMC13055307; doi:10.1128/spectrum.02135-25)
Supplement: Table S2 — Sequence similarity analysis of putative novel viruses' aa sequence from seven families identified by meta-viromic sequencing for mosquitoes in Egypt. [file spectrum.02135-25-s0008.docx]

**Table S2.** Sequence similarity analysis of putative novel viruses’ aa sequence from seven families identified by meta-viromic sequencing for mosquitoes in Egypt.

| Family | Novel virus | Strain | Sequence* (Genbank ID) | Amino acid (aa) | |
| --- | --- | --- | --- | --- | --- |
|  |  |  |  | gene | aa identity (%) |
| *Amalgaviridae* | Qalyubia amalga-like virus 1 | EG01 | Sanya amalgavirus 1 (MZ209909) | RdRp | 35 |
| *Chrysoviridae* | Egypt chrysovirus-like virus | EG01, EG02, EG03 | Raphanus sativas chrysovirus 1 (NC_043657) | RdRp | 43 |
| *Mitoviridae* | Egypt mitovirus-like virus 1 | EG01 | Chronajilt virus (PP172240) | RdRp | 35 |
|  | Egypt mitovirus-like virus 2 | EG01 | Mitoviridae sp. (ON163950) | RdRp | 34 |
| *Totiviridae* | Egypt totivirus-like virus 1 | EG01, EG02 | Culex vishnui subgroup totivirus (LC514295) | RdRp | 83 |
|  | Egypt novel toti-like virus 1 | EG01, EG02 | XiangYun toti-like virus 11 (OL700200) | Capsid protein | 31 |
|  |  | EG03, EG04 | XiangYun toti-like virus 11 (OL700200) | RdRp | 37 |
| *Virgaviridae* | Egypt associated virga-like virus 1 | EG01 | Plasmopara viticola lesion associated virga-like virus 1 (MN551100) | Replication protein | 36 |
|  | Egypt obamo-like virus 1 | EG01 | Nigrospora aurantiaca tobamo-like virus 1 (OR228589) | Replication protein | 69 |
| *Narnaviridae* | Egypt Narnaviridae sp 1 | EG01 | Narnaviridae sp (ON164222) | RdRp segment | 65 |
| *Orthomyxoviridae* | Egypt associated orthomyxo-like virus 1 | EG01 | XiangYun orthomyxo-like virus 2 (OL700154) | RdRp segment | 64 |

*Sequences most closely related to putative novel virus’s genome sequences and amino acid sequences in phylogenetic analyses.
