# Supplementary material for: Metagenomic sequencing reveals viral diversity of mosquitoes from Egypt: co-circulation of multiple insect-specific viruses
Source: Microbiol Spectr. 2026 Mar 6;14(4):e02135-25. doi: 10.1128/spectrum.02135-25 (PMC13055307; doi:10.1128/spectrum.02135-25)
Supplement: Fig. S1 — Map of Egypt (survey sites are shown on map). [file spectrum.02135-25-s0001.pdf]

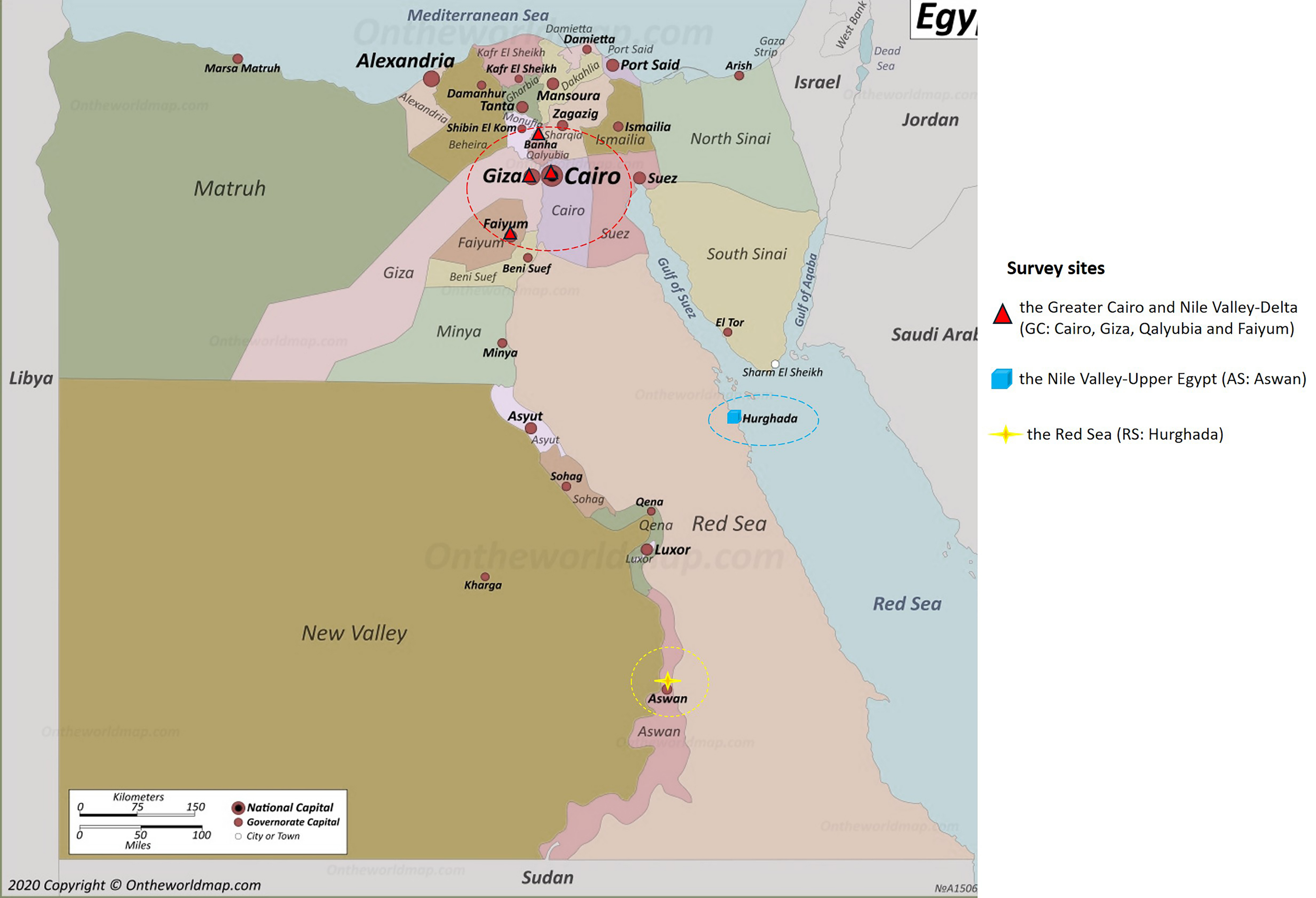
Egy
Mediterranean Sea
Damietta
Damietta
Port Said
Port Said
Gaza Strip
West Bank
Dead Sea
Israel
Jordan
Arish
Alexandria
Marsa Matruh
Kafr El Sheikh
Kafr El Sheikh
Damanhur
Gharbia
Dakahlia
Mansoura
Tanta
Alexandria
Monufia
Zagazig
Ismailia
Shibin El Kom
Sharqia
Ismailia
North Sinai
Beheira
Banha
Qalyubia
Giza
Cairo
Suez
Matruh
Cairo
Faiyum
Faiyum
Suez
South Sinai
Beni Suef
Beni Suef
Giza
Gulf of Suez
Gulf of Aqaba
El Tor
Minya
Minya
Saudi Arab
Libya
Sharm El Sheikh
Asyut
Hurghada
Asyut
Sohag
Sohag
Qena
Qena
Red Sea
Luxor
Luxor
Kharga
Red Sea
New Valley
Aswan
Aswan
Kilometers
0 75 150
0 50 100
Miles
National Capital
Governorate Capital
City or Town
Sudan
2020 Copyright © Ontheworldmap.com
№A1506
Survey sites
the Greater Cairo and Nile Valley-Delta (GC: Cairo, Giza, Qalyubia and Faiyum)
the Nile Valley-Upper Egypt (AS: Aswan)
the Red Sea (RS: Hurghada)
